# Socioeconomic impacts of airborne and droplet-borne infectious diseases on industries: a systematic review

**DOI:** 10.1186/s12879-024-08993-y

**Published:** 2024-01-16

**Authors:** Ely Zarina Samsudin, Siti Munira Yasin, Nur-Hasanah Ruslan, Nik Nairan Abdullah, Ahmad Faiz Azhari Noor, Ahmad Fitri Abdullah Hair

**Affiliations:** 1 Department of Public Health Medicine, Faculty of Medicine, Universiti Teknologi MARA, Sungai Buloh, Malaysia; 2 Faculty of Sports Science and Recreation, Universiti Teknologi MARA, Shah Alam, Malaysia; 3 Occupational Health Division, Department of Occupational Safety and Health, Putrajaya, Malaysia

**Keywords:** Infectious disease, Outbreak, Epidemic, Pandemic, Socioeconomic impact, Socioeconomic burden, Socioeconomic cost

## Abstract

**Background:**

Recent pandemics have had far-reaching effects on the world’s largest economies and amplified the need to estimate the full extent and range of socioeconomic impacts of infectious diseases outbreaks on multi-sectoral industries. This systematic review aims to evaluate the socioeconomic impacts of airborne and droplet-borne infectious diseases outbreaks on industries.

**Methods:**

A structured, systematic review was performed according to the PRISMA guidelines. Databases of PubMed, Scopus, Web of Science, IDEAS/REPEC, OSHLINE, HSELINE, and NIOSHTIC-2 were reviewed. Study quality appraisal was performed using the Table of Evidence Levels from Cincinnati Children’s Hospital Medical Center, Joanna Briggs Institute tools, Mixed Methods Appraisal Tool, and Center of Evidence Based Management case study critical appraisal checklist. Quantitative analysis was not attempted due to the heterogeneity of included studies. A qualitative synthesis of primary studies examining socioeconomic impact of airborne and droplet-borne infectious diseases outbreaks in any industry was performed and a framework based on empirical findings was conceptualized.

**Results:**

A total of 55 studies conducted from 1984 to 2021 were included, reporting on 46,813,038 participants working in multiple industries across the globe. The quality of articles were good. On the whole, direct socioeconomic impacts of Coronavirus Disease 2019, influenza, influenza A (H1N1), Severe Acute Respiratory Syndrome, tuberculosis and norovirus outbreaks include increased morbidity, mortality, and health costs. This had then led to indirect impacts including social impacts such as employment crises and reduced workforce size as well as economic impacts such as demand shock, supply chain disruptions, increased supply and production cost, service and business disruptions, and financial and Gross Domestic Product loss, attributable to productivity losses from illnesses as well as national policy responses to contain the diseases.

**Conclusions:**

Evidence suggests that airborne and droplet-borne infectious diseases have inflicted severe socioeconomic costs on regional and global industries. Further research is needed to better understand their long-term socioeconomic impacts to support improved industry preparedness and response capacity for outbreaks. Public and private stakeholders at local, national, and international levels must join forces to ensure informed systems and sector-specific cost-sharing strategies for optimal global health and economic security.

**Supplementary Information:**

The online version contains supplementary material available at 10.1186/s12879-024-08993-y.

## Background

For every country across the globe, the industries and sectors have fundamental roles in both its economic and social development. Not only are industries a main contributor to a country’s gross domestic product (GDP) and economic growth, it is critical for employment creation, technological advancements, and general improvements in living standards. In 2021, the services, manufacturing, agriculture, forestry, and fishing industries contribute to 65.7, 28.3, and 4.3% of the world GDP and accounts for 51, 23, and 27% of total employment, respectively [1]. Over the past decades, industrialisation and the accompanying economic growth in terms of increase in per capita GDP have resulted in increases in wages and household incomes, as well as improved nutrition, housing, sanitation, medical care, and literacy [2, 3].

Despite the era of modernization and public health advances, regional and global emerging and endemic infectious diseases outbreaks continue to not only adversely impact global health systems, but also give rise to wider socioeconomic consequences [4]. This includes airborne and droplet-borne infectious diseases incidences of varying scale and magnitude, including endemics, outbreaks, epidemics, and pandemics. The ongoing pandemic of Coronavirus Disease 2019 (COVID-19), declared a global emergency by the World Health Organisation (WHO) on January 30, 2020, has had far-reaching impacts on the world’s largest economies, including industries of the primary, secondary, and tertiary sectors [5]. These include increased healthcare costs, job losses, macroeconomic instability, and dwindling in micro, small, medium-sized enterprises (MSME) as well as informal industries [5, 6]. Health disasters such as the Severe Acute Respiratory Syndrome (SARS) pandemic in 2003, which lasted approximately 6 months, had led to a total global economic loss of approximately USD40 billion due to its impacts on the hospitality, commerce, transport and multi-national industries such as the oil industry [4]. Similarly, the influenza A (H1N1) 2009–2010 pandemic led to severe economic recession and crash in the stock market values of multiple industries [7].

These socioeconomic effects can be felt not only from large-scale infectious diseases outbreaks, but from outbreaks of a smaller scale as well. Seasonal influenza epidemics continue to pose direct and indirect costs to organisations, including absenteeism, losses in productivity, and impaired performance [8]. Norovirus outbreaks, a common occurrence in semi-enclosed settings, has led to a loss of USD2 billion in the United States of America (USA) alone, due to lost productivity and healthcare expenses [9]. Meanwhile, endemic infectious diseases such as tuberculosis adversely affects the labour force, disrupts local economies, and is projected to result in an economic loss of USD17.5 trillion based on estimations of tuberculosis mortality from 2020 to 2050 in 120 countries [10]. Evidence suggests that respiratory pathogens such as severe acute respiratory syndrome coronavirus 2 (SARS-CoV-2), severe acute respiratory syndrome coronavirus (SARS-CoV), and influenza virus have airborne transmission, culminating in numerous superspreading events that led to the spread of these diseases at alarming rates and causing huge devastations on global economies [11].

The escalating costs associated with airborne and droplet-borne infectious diseases have amplified the need to estimate the full extent and range of socioeconomic impacts on multi-sectoral industries. A greater appreciation of these impacts would enable an assessment of burden of diseases as well as contribute towards the development of long-term prevention and preparedness measures, prioritization exercises, and optimization of resources. Unfortunately, there is presently limited evidence for the socioeconomic impacts of infectious diseases on industries. Previous studies that have explored this subject were studies focusing on particular geographical regions [12, 13] or specific infectious diseases such as COVID-19 [14, 15], influenza [8, 16, 17], and tuberculosis [10], or studies examining economic impacts exclusively [18, 19]. On the other hand, Smith et al. (2019) [4] illustrated the multi-sectoral socioeconomic impacts of infectious diseases using a case-study approach, but the findings relate to pre-COVID-19 pandemic era. With this in mind, this study aims to systematically examine the pool of evidence pertaining socioeconomic impacts of airborne and droplet-borne infectious diseases on industries and conceptualizing a framework based on empirical findings.

## Methods

This systematic review was reported in accordance to the Preferred Reporting Items for Systematic Reviews and Meta-Analyses (PRISMA) guidelines [20]. The systematic review protocol was registered in INPLASY Register (Registration No. INPLASY202190055).

### Design and research aims

A structured, systematic review and qualitative synthesis of peer-reviewed publications was performed to explore the socioeconomic and safety and health impacts of airborne and droplet-borne infectious diseases in industries; however, due to the high numbers of included studies this review will be focused on socioeconomic impacts exclusively. Due to the heterogeneity of included studies, quantitative analysis was not attempted.

### Search strategy

A comprehensive search of the literature was undertaken in August 2021 using three biomedical electronic database (PubMed, Scopus, and Web of Science), one economic database (IDEAS/REPEC) and three occupational safety and health databases (OSHLINE, HSELINE, and NIOSHTIC-2). The search aimed to identify relevant articles published in peer-reviewed journals written in English, with the assumption that most of the important findings will be reported in English regardless of country of origin. Boolean search was performed on each database, without restriction to date or publication, as illustrated in Supplementary Document 1.

The terms included in the Boolean search were chosen after careful consideration and consensus of terms identified from literature review, in view of the variation in keywords of interest. The first combination of keywords included various terms denoting socioeconomic and occupational safety and health impacts of infectious diseases at the workplace as described by previous studies [4, 21–24]. The second combination of keywords included key terms related to infectious diseases and common pathogens that may spread via droplets and airborne transmission [25]. Herein, droplet-borne infectious disease was defined as an infectious disease which is transmitted when a person is exposed to infective respiratory droplets, whereas airborne infectious disease was defined as an infectious disease which is transmitted when a person is exposed to droplet nuclei (aerosols) [26]. Finally, the third combination of keywords included terms that specify workplace settings. To broaden the search, the Boolean search operator “OR” was used with multiple analogous terms, whereas “AND” was used to narrow the search to studies examining socioeconomic and safety and health impacts of infectious diseases on workers in industries. The search was conducted by one reviewer. All searches were concluded by 29th August 2021.

### Selection criteria and study selection

Upon completion of the searches, articles were organized into EndNote 20 Software. Duplicates were identified and removed. This was performed by one reviewer, firstly using the “Find and Remove Duplicate References” function, and secondly using manual screening given that a number of the same articles were entered differently into different databases. Following duplicates removal, articles were assessed for eligibility independently by two reviewers in two stages. In stage one, the title and abstract of search results were screened and assessed for relevance. In stage two, the full-text of potentially relevant publications were retrieved and reviewed for inclusion. Any primary studies in English examining socioeconomic impacts of airborne and droplet-borne infectious diseases outbreaks in any industry were included. Here, socioeconomic impacts in industries was defined as impacts related to social and economic aspects of industries, such as the morbidity and mortality, costs associated with disease diagnosis, treatment, and prevention, as well as productivity loss, employment, financial loss, and disruption in supply chain and services [7, 27]. Non-human studies, non-primary studies including reviews, editorials, commentaries, forewords, opinion pieces, and books, studies that examined infectious diseases transmitted via routes other than airborne and droplet-borne transmission, studies examining variables others than socioeconomic impacts, and studies not concerning industries or workers were excluded. The reason for excluding a publication following title and abstract review as well as full-text review was noted. Based on the inclusion and exclusion criteria described previously, the list of studies included and excluded was cross-validated. Consensus was obtained where possible for any disagreement, and in cases when not, and a third reviewer was assigned. The per cent agreement and Cohen’s Kappa were 99.8% and 0.993 respectively for stage one and 98.4% and 0.955 respectively for stage two of the study selection process, which indicated excellent interrater reliability [28]. To allow for quality assessment, measures to contact authors for articles not available in full text were taken, and only full text articles were included in the review. Due to resource limitations, hand searching was not performed.

### Quality assessment

The quality of included studies was examined by evaluating the level of evidence according to the Table of Evidence Levels from Cincinnati Children’s Hospital Medical Center (CCHMC) [29] and quality of study according to the Joanna Briggs Institute (JBI) tools [30] and Center of Evidence Based Management (CEBMa) case study critical appraisal checklist [31] (Supplementary Document 2). The CCHMC classifies level of evidence for individual studies by study design, domain, and quality, with level 1 representing the highest level and indicating the strongest evidence, and level 5 representing the lowest level and indicating the weakest evidence [29]. In addition, the JBI and CEBMa tools were used to further subclassify studies at each level to either “a” or “b”, which signifies good quality and lesser quality study respectively in terms of methodological quality. The JBI tools are widely used critical appraisal tools developed by the Joanna Briggs Institute, a researching and development organisation based in the Faculty of Health and Medical Sciences at the University of Adelaide, South Australia [30]. Compared to other tools, the applicable range of the JBI tools are wide and they are deemed to be highly coherent appraisal instruments [32, 33]. On the other hand, the CEBMa tools were developed by CEBMa [31] for assessing the methodological quality of case studies. Both JBI and CEBMa tools include critical appraisal checklists for specific study designs. For longitudinal studies, the JBI tool for cohort studies was applied, and the ratings related to Question 1, 2 and 6 which are specific for cohort study were marked as not applicable. Based on a ‘star system’, a star was awarded for every quality criterion met by the study and the quality rating was assigned as follows:

- Longitudinal studies: 8 maximum stars and a final rating of 0–2 stars as “poor”, 3–4 stars as “moderate”, 5–6 stars as “good” and 7–8 stars as “excellent”
- Cohort studies: 11 maximum stars and a final rating of 0–2 stars as “poor”, 3–5 stars as “moderate”, 6–8 stars as “good” and 9–11 stars as “excellent”
- Case-control studies: 10 maximum stars and a final rating of 0–2 stars as “poor”, 3–5 stars as “moderate”, 6–7 stars as “good”, and 8–10 stars as “excellent”
- Analytical cross-sectional studies: 8 maximum stars and a final rating of 0–2 stars as “poor”, 3–4 stars as “moderate”, 5–6 stars as “good”, and 7–8 stars as “excellent”
- Prevalence studies: 9 maximum stars and a final rating of 0–2 stars as “poor”, 3–5 stars as “moderate”, 6–7 stars as “good”, and 8–9 stars as “excellent”
- Qualitative studies: 10 maximum stars and a final rating of 0–2 stars as “poor”, 3–5 stars as “moderate”, 6–7 stars as “good”, and 8–10 stars as “excellent”
- Case studies: 10 maximum stars and a final rating of 0–2 stars as “poor”, 3–5 stars as “moderate”, 6–7 stars as “good”, and 8–10 stars as “excellent”

In the final quality rating, studies under the categories “excellent” and “good” were rated as “a” and those under the categories “poor” and “moderate” were rated as “b”. The quality assessment was performed independently by two reviewers. Data extraction and analysis were cross-validated to assess for disagreements. For any disagreement that was present, consensus was sought where possible. A third reviewer was assigned in cases where that were not possible.

### Data extraction and analysis

For each of the included study, data on author, year of publication, location of study, industry, type of infectious disease, year of outbreak, study design, study population, number of participants included, study variables examined, study instruments used, and socioeconomic impacts were extracted. Using the web-based tool CCEMG – EPPI-Centre Cost Converter (v.1.6), all estimates of costs was converted to US dollars (USD) for consistency based on the International Monetary Fund source dataset for purchasing power parity values and same base-year as reported in the original study [34]. The data extraction was performed independently by two reviewers. For any disagreement that was present, consensus was sought where possible, and in cases where that were not possible, a third reviewer was assigned. Data was analysed qualitatively due to the heterogeneity of studies included in the systematic review, and meta-analysis was not attempted. Where applicable, data was analysed using descriptive statistics using Statistical Package of Social Science Version 27. The numerical data was analysed using mean and standard deviation, while the categorical data was analysed using frequency and percentage.

## Results

### Study characteristics and methodological quality of studies

**Fig. 1:**
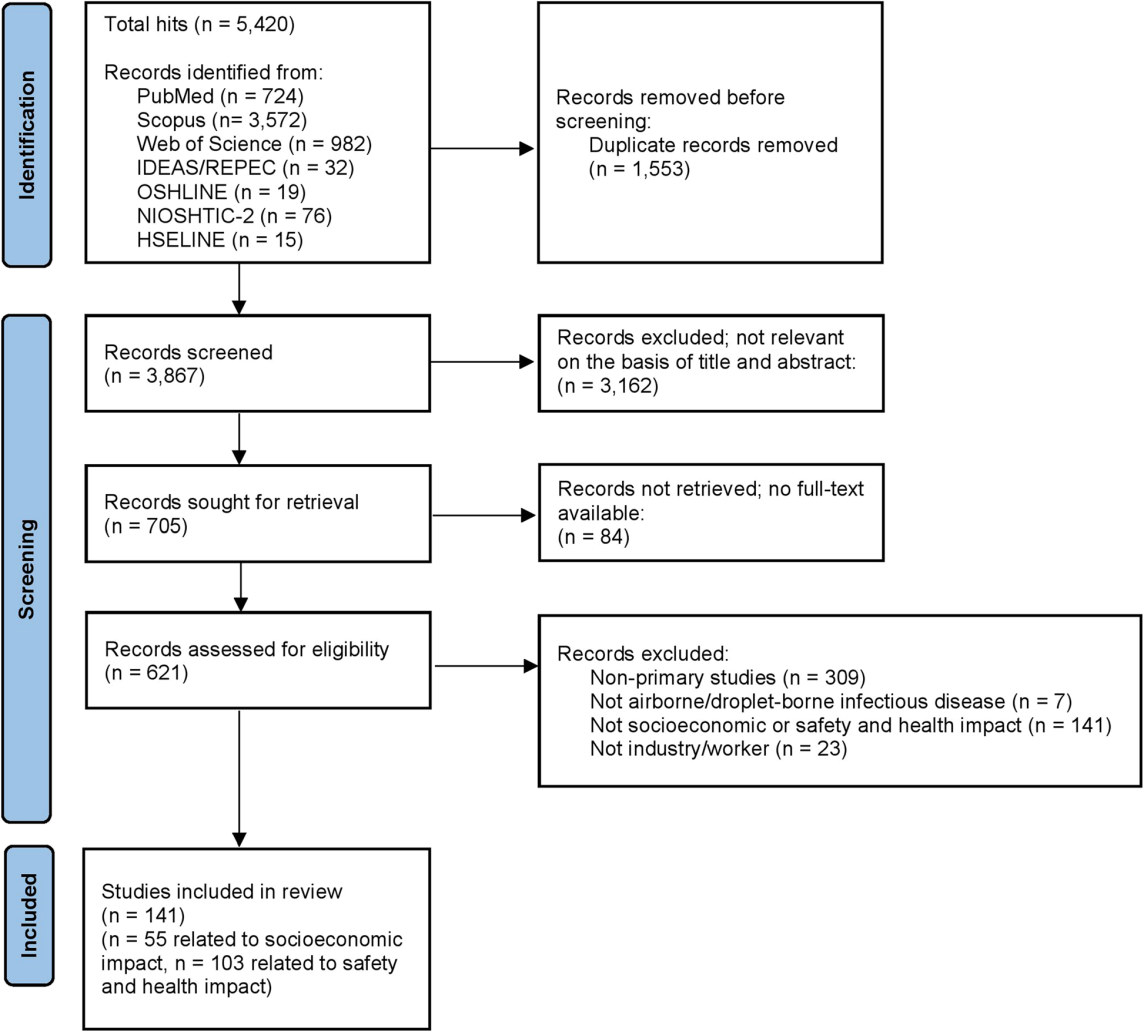
Flow diagram of the systematic review based on the PRISMA statement

A total of 5420 articles were initially identified, and after removing duplicates, 3867 articles were screened. 3162 articles were excluded due to not being relevant on the basis of title and abstract. 84 articles were then excluded due to full-text non-availability. 480 articles did not meet the inclusion criteria, and a total of 141 articles were finally included. Of those, 55 studies were related to socioeconomic impact and were thus included in this review. The flow chart of the study search and selection is illustrated in Fig. 1, using the PRISMA format.

The summary of the studies included in this systematic review can be found in Table 1. The studies were published from 1984 to 2021, and were conducted in all parts of the world, including countries from North America (44%), South America (6%), Europe (24%), Asia (13%), Africa (4%), and Australasia (7%) regions, as well as globally (2%). Majority of studies (47%) were related to the healthcare industry, followed by multiple (31%), hospitality (5%), education (4%), transport (4%), agriculture (4%), construction (2%), chemical (2%), and commerce industries (2%). In terms of types of airborne or droplet-borne infectious diseases examined, the vast majority (62%) studied COVID-19, whereas 24% studied influenza (24%), followed by influenza A (H1N1) (9%), SARS (4%), tuberculosis (2%) and norovirus (2%).

**Table 1:** Summary of included studies examining socioeconomic impact of airborne and droplet-borne infectious disease on industries

Author (Year) / Location	Industry	Type of Infectious Disease	Year(s) Outbreak/Epidemic/Pandemic Occurred	Study Design	Study Population (*N*)	Study Variables	Study Instruments/Method	Socioeconomic Impact
Akazawa et al. (2003) [35] / USA	Multiple	Influenza	1996	Cross-sectional study	Workers across USA (*N* = 7037)	Absenteeism, work loss	1996 Medical Expenditure Panel Survey data modelling	1. The average number of workdays missed due to ILI was 1.30 days 2. The average work loss was valued at USD137 per person
Al-Ghunaim et al. (2021) [36] / UK	Health-care	COVID-19	2020-current	Qualitative study	Surgeons across UK (*N* = 141)	Productivity, employee engagement	Qualtrics survey tool and thematic analysis	1. Participants reported being less productive and slower at work 2. Participants reported decreased motivation levels at work
Alsharef et al. (2021) [37] / USA	Con-struction	COVID-19	2020-current	Qualitative study	Professional organizations subject matter experts (*N* = 34)	Supply chain disruption, supply cost, production cost, service disruption, employment, workforce size, productivity, absenteeism, demand shock	Semi-structured interview	1. Containment measures included provision of temporary shutdown and quarantining, PPE, and COVID-19 related training 2. Material shortages and material price escalation 3. Delays in material delivery, which caused significant schedule disruptions 4. Increased production cost due to need to offer larger compensations to subcontractors and additional cost and overhead 5. Large number of furloughs and layoffs due to cash flow challenge and workload reduction 6. Reduced workforce due to social distancing recommendations and absenteeism 7. Loss in productivity and efficiency 8. Increased demand for home improvement and renovation products and supplies from local supplier and manufacturers
Banerjee et al. (2021) [38] / global	Health-care	COVID-19	2020-current	Longi-tudinal study	Oncology professionals from 101 countries (*N* = 1520 survey I, *N* = 272 survey I & II)	Productivity	Job Performance since COVID-19 tool	1. 49% participants reported that they were unable to do their job to the same standard compared with pre-COVID-19 period
Bergeron et al. (2006) [39] / Canada	Health-care	SARS	2003	Cross-sectional study	Community nurses across Ontario (*N* = 941)	Workforce size, service disruption	Self-administered questionnaire and thematic analysis	1. Containment measures included patient and visitor screening, and mandatory PPE 2. 66% participants cited staff shortages and program stoppages
Brophy et al. (2021) [40] / Canada	Health-care	COVID-19	2020-current	Qua-litative study	Frontline HCW across Ontario (*N* = 10)	Workforce size, absenteeism	In-depth interview and thematic analysis	1. Containment measures included PPE and sanization protocols 2. Increased staff shortages 3. Increased absenteeism among frontline HCW
Calvo-Bonacho et al. (2020) [41] / Spain	Multiple	COVID-19	2020-current	Cross-sectional study	Workers covered by Spanish insurance company (*N* = 1,651,305)	Absenteeism, work loss	National Public Health System Register data analysis	1. Dramatic increase of sick leave for respiratory diseases and infectious disease in March 2020 (4.9 cases vs 2.5 cases and 5.1 cases vs 1.3 cases per 1000 workers in 2020 vs. 2017, 2018, and 2019 respectively), representing 116% increase in total sick leave 2. The increased sick leave translated into an 40% increase of associated costs during the first trimester of 2020 compared with the same period of 2017–2019 (mean USD4374.81 vs. 3118.20 per 100 affiliated workers)
Caroll & Smith (2020) [42] / USA	Health-care	COVID-19	2020-current	Case study	Hospital in Washington State (*N* = 1)	Financial loss, supply cost	2019 hospital financial data analysis	For duration of shift for 3-months: 1. 25–50% reduction in surgical volume resulting in USD12.46–24.9 million revenue loss, and 10–20% reduction in clinic volume resulting in USD0.64–1.29 million loss 2. Increase of USD107,040–535,198 in supply costs 3. Loss of USD124,480 (1% increase in ICU days) to a loss of USD2.49 million (20% increase in ICU days) due to substitution of acute care for critical care Per year: 1. Estimated financial loss of USD13–117 million/year
Challener et al. (2021) [43] / USA	Health-care	Influenza	2009–2019	Case study	Large academic medical centre workforce	Presenteeism, absenteeism	2009–2019 biweekly institutional payroll data analysis	1. ILI is a statistically significant predictor of unscheduled absences in both salaried and hourly workers (*p* < 0.01) 2. For every increase in ILI by 1% in the population of the state, hourly workers and salaried workers have an increase in percent of unscheduled absence hours of 0.14 and 0.04% respectively 3. For every increase in ILI by 1%, the proportion of paid hours that are worked increases by 0.2% (*p* = 0.04) in hourly workers
Considine et al. (2011) [44] / Australia	Health-care	H1N1 flu	2009	Cross-sectional study	EM and nursing staff across Australia (*N* = 618)	Absenteeism	Self-developed survey	1. 35% participants reported ILI; the mean number of days away from work due to ILI was 3.73 (SD = 3.63)
Danial et al. (2016) [45] / Scotland	Health-care	Norovirus	2013	Case study	Hospital in Scotland (*N* = 1)	Work loss, healthcare cost	APEX system data analysis and economic analysis	In the outbreak which occurred from January until March of 2013: 1. 30 HCW (3.10 cases per 1000 inpatient bed-days) were affected, i.e. developed gastroenteritis 2. Total cost of staff absence was USD16,232.42 3. Healthcare cost included loss due to empty beds (USD401,893.83), cleaning costs (USD5,021.52), incident management team (USD64,562.41), and laboratories (USD2,295.55)
Delaney et al. (2021) [46] / USA	Health-care	COVID-19	2020-current	Cross-sectional study	University of Utah staff (*N* = 5030)	Employee engagement, productivity	Self-developed survey	1. 21% participants moderately or very seriously considered leaving the workforce and 30% considered reducing hours 2. 39% participants felt their productivity decreased
Duarte et al. (2017) [47] / Chile	Health-care	Influenza	2009	Cross-sectional study	Employed, privately insured Chileans (*N* = 1.4 M)	Absenteeism, workforce size	Private health insurance claim data analysis	1. Pandemic increased mean flu days missed by 0.042 days per person-month during the 2009 peak winter months (June and July), representing an 800% increase in missed days 2. Minimum of 0.2% reduction in Chile’s labour supply was observed
Escudero et al. (2005) [48] / Singapore	Health-care	SARS	2003	Cross-sectional study	Tan Tock Seng Hospital HCW (*N* = 4261)	Absenteeism	Surveillance data for staff on sick leave analysis	1. Containment measures included surveillance 2. 4261 staff as of mid-Sept 2003 had episodes of staff MC for febrile illness 3. The rate of staff sick leave for febrile illness was 1.40 per 1000 staff-days observed 4. There were 57 cases of deaths with pneumonia
Fargen et al. (2020) [49] / USA	Health-care	COVID-19	2020-current	Cross-sectional study	Neurointerven-tional physician organization members (*N* = 151)	Employment	Self-developed survey	1. COVID-19-positive infections occurred in 1% of respondents, and an additional 8% were quarantined for suspected infection. 2. 1% participants reported their employment position being terminated or furloughed 3. 30 and 23% participants reported reduction of 25% or less and greater than 25% of normal compensation respectively
Gashi et al. (2021) [50] / Kosovo	Multiple	COVID-19	2020-current	Cross-sectional study	Micro, small, medium and large business enterprise workers (*N* = 205)	Employment, financial loss, supply chain disruption	Self-developed survey	1. National containment measures included lockdown, closure of borders, and restriction of free movement 2. 37% participants had laid off employees: 25% have laid off 1–4 employees, 8% 5–10 employees, and 3% 11–90 employees 3. On average, microenterprises incurred losses of USD32,643.53, small enterprises USD316,624.61, medium enterprises USD804,205.05, and large companies USD864,353.31 4. 90% participants (40.5% greatly, 28% somewhat, and 22% a little) reported that they were affected by supply of materials
Gray et al. (2021) [51] / USA	Health-care	COVID-19	2020-current	Longi-tudinal study	Critical care physicians across US (*N* = 2375 T0, *N* = 1356 T1)	Workforce size	Self-developed survey	1. Substantial shortages of ICU-trained staff reported in T0, although declining slightly, persist in T1; 48% in T0 vs. 47% in T1 2. The largest staffing shortage reported for both T0 and T1 was in ICU-trained nurses (34% in T0 vs. 33% in T1)
Groenewold et al. (2013) [52] / USA	Multiple	H1N1 flu	2009	Cross-sectional study	Full-time US workers (*N* = 60,000 households)	Absenteeism	Current Population Survey database analysis	1. There was a significant (*p* < .01) increase in health-related absenteeism among full-time workers above baseline, corresponding with pandemic peak in national occurrence of ILI 2. Total one-week absenteeism ranged from 2 to 4% 3. Peak workplace absenteeism was correlated with the highest occurrence of both ILI and influenza-positive laboratory tests
Groenewold et al. (2019) [53] / USA	Multiple	Influenza	2017–2018	Cross-sectional study	Full-time US workers (*N* = 60,000 households)	Absenteeism	Current Population Survey database analysis	1. Prevalence of health-related work absenteeism among full-time workers peaked at 3.0% (95% CI 2.8–3.2%) in January 2018 2. Regional absenteeism peaks corresponded to concurrent peaks in ILI activity
Groenewold et al. (2020) [54] / USA	Multiple	COVID-19	2020-current	Cross-sectional study	Full-time US workers (*N* = 60,000 households)	Absenteeism	Current Population Survey database analysis	1. In March and April 2020, prevalence of health-related workplace absenteeism among all full-time workers estimates exceeded the epidemic threshold 2. In April 2020, absenteeism among the following occupational subgroups significantly exceeded their occupation-specific epidemic thresholds: personal care and service (include childcare workers and personal care aides) (5.1% [95% CI = 3.5–6.7]), healthcare support (5.0% [95% CI = 3.1–6.8], production (3.7% [95% CI = 2.7–4.7], transportation and material moving occupations (include bus drivers and subway and streetcar workers) (3.6% [95% CI = 2.6–4.6], and healthcare practitioner and technical occupations (2.8% [95% CI = 2.0–3.6]
Haidari et al. (2021) [55] / USA	Health-care	COVID-19	2020-current	Cross-sectional study	California Mat-ernal/Perinatal Quality Care Collaborative webinar atten-dees (*N* = 288)	Work error, productivity	Self-developed survey	1. 12% participants reported increased medical errors 2. 59% participants reported difficulty meeting home and work responsibilities
Hammond & Cheang (1984) [56] / USA	Health-care	Influenza	1980–1981	Cross-sectional study	Winnipeg Health Sciences Centre hospitals HCW (*N* = 1600)	Absenteeism, work loss	Hospital records data analysis	1. Comparisons between the peak 2-week periods of absenteeism during the epidemic and baseline “control” period showed increase in absenteeism rate during the epidemic (0.0586 vs 0.0346) 2. The total salary paid out for sick leave in the 2-week period of peak absenteeism during the epidemic was much greater than that paid out in the comparable period the next year when no influenza epidemic occurred (USD60,776.13 vs. USD36,290.00)
Harrop et al. (2021) [57] / USA	Education	COVID-19	2020-current	Cross-sectional study	US early career university researchers (*N* = 150)	Productivity	Self-developed survey	1. 85% participants reported a loss of productivity compared to “normal”, with the majority reporting they were currently working between 41 and 60% (33% participants) or 61–80% (38% participants) productivity
Hasan et al. (2021) [58] / Bangladesh	Agri-culture	COVID-19	2020-current	Cross-sectional study	Farmers, middlemen and consumers across Dhobaura (*N* = 280)	Production cost, financial loss, employment, demand shock	Self-developed survey	1. The total production costs (primary fixed costs, operation costs, feed costs, medicinal costs) has increased since the pandemic and gross margins reduced 2. To reduce labour costs, 80% of farms reduced staff; the number of staff employed decreased from 209 to 149 following the pandemic (median change − 1.5). Overall mean labor cost/day dropped from USD 3.93 to USD 3.52 (> 10% fall) 3. The finfish farmers were receiving less profits, suffering a real price reduction of USD0.16/kg. By contrast, the middlemen have increased their selling prices, presumably to offset increased costs and maintain profitability 4. Small decrease in demand for dish; fish main protein source for 85% respondents pre-COVID, which dropped to 64.2% after the pandemic, and the amount of fish purchased decreased with a reduction in consumers buying over 5 kg from 46.7 to 30% between pre- and post-COVID
Hemmington & Neill (2021) [59] / New Zealand	Hospitality & Tourism	COVID-19	2020-current	Cross-sectional study	Senior industry executives from 105 restaurants, café, take-away outlet (*N* = 11)	Financial loss, demand shock, production cost, employment, business disruption	Self-developed qualitative survey	1. National containment measures: COVID-19 Alert Level 1–4 2. No tourism and no large concert gatherings led to lower demand 3. As COVID-19 level rose, café incomes declined 4. As customers decreased, there was increased “spend per head” 5. Many staff laid off 6. Operators with low margins and poor cashflow have gone out of business
Iacus et al. (2020) [60] / Italy	Transport	COVID-19	2020-current	Cross-sectional study	Global aviation sector	Demand shock, GDP loss, work loss	Forecasting modelling based on past pandemic crisis and observed flight volumes	1. Travel ban imposed since start of pandemic led to demand shock 2. At the end of 2020 the GDP loss globally could be as high as 1.41–1.67% and job losses may reach the value of 25–30 millions in the worst case scenarios 3. Focusing on EU27, the GDP loss may amount to 1.66–1.98% by the end of 2020 and the number of job losses from 4.2 to 5 million in the worst case scenarios
Jazieh et al. (2021) [61] / Middle-east, North Africa, Brazil, Phillipines	Health-care	COVID-19	2020-current	Cross-sectional study	Middle-east, North Africa, Brazil, Phillipines oncologists (*N* = 1010)	Productivity	Self-developed survey	1. 3% of participants contracted COVID-19 infection 2. 34% participants reported negative pandemic impact on their research productivity
Jha et al. (2020) [62] / USA	Health-care	COVID-19	2020-current	Cross-sectional study	American Society of Interventional Pain Physicians members (*N* = 100)	Employment, financial loss, employee engagement, business disruption	Self-developed survey	1. 56% participants have reduced staffing through furloughs and/or layoffs, and 68% have reduced hours per staff 2. 91% participants have seen reduction in collections 3. 55% participants reported that feelings of burnout have made them want to quit practicing medicine 4. 19% participants reported having had to close office
Jiménez-Labaig et al. (2021) [63] / Spain	Health-care	COVID-19	2020-current	Cross-sectional study	Spanish oncology doctors (*N* = 243)	Employee engagement	Self-developed survey	1. 17% participants reported having been infected with SARS-CoV-2 2. 23% participants reported doubts about their medical vocation
Jones et al. (2021) [64] / USA	Health-care	COVID-19	2020-current	Cross-sectional study	US health system pharmacists (*N* = 484)	Employment	Self-developed survey	1. 1, 6, and 17% participants reported having lost their job, being furloughed, and decreased salary respectively
Karatepe et al. (2021) [65] / Turkey	Hospitality & Tourism	COVID-19	2020-current	Cross-sectional study	2 Turkish national 5-star hotels worker (*N* = 150)	Absenteeism	Autry & Daugherty (2003) absenteeism item	1. COVID-19 pandemic significantly associated with absenteeism among participants (*p* < 0.01)
Karve et al. (2013) [66] / USA	Multiple	Influenza	2000–2009	Cross-sectional	MarketScan CCAE and HPM database workers (*N* = 40 M)	Healthcare cost, work loss	2 MarketScan databases 2000–2009 data analysis	1. The average per-patient influenza-related medical cost (ILI-related medical, inpatient, outpatient, physician office, emergency department, pharmacy, ancillary care utilization and costs) ranged from USD239.43 to USD300.83 2. 30% participants with influenza diagnosis had > 1 day of influenza-related work absence during the nine influenza seasons studied 3. The average per-patient cost associated with influenza-related work absence, across all seasons studies, was USD209.66 4. The cost of average influenza-related productivity losses per 100,000 plan members, across all seasons studied, was USD42.58
Keech et al. (1998) [67] / UK	Chemical	Influenza	1994–1995	Longi-tudinal study	Large UK pharmaceutical company workers (*N* = 411)	Absenteeism, presenteeism, work loss, healthcare cost	Self-developed questionnaire and diary card	1. The mean number of missed workdays was 2.8 days, with means ranging from 3.2 days for secretarial/administrative staff to 1.8 days for managers 2. For those who returned to work while symptomatic, 81% felt only moderately effective 3. 73% participants reported that the illness had interfered with work in or around the home ‘all or most of the time’ 4. Overall total cost of missed work days was an estimated USD159,769.67 5. Overall healthcare cost (pharmacist consultation, GP visits and consultations, ED visits, hospitalization, medication) for participants was estimated USD2,512.16
Lee et al. (2008) [68] / Hong Kong	Hospitality & Tourism	Influenza	2007	Cross-sectional study	Hong Kong corporation staff (*N* = 2212)	Work loss, productivity	Self-developed survey	1. Average equivalent days of perfect health loss per person per year was 10.71 days 2. Average productivity loss per person per year was USD152.12
Leigh (2011) [69] / USA	Multiple	Tuber-culosis	2007	Longi-tudinal study	Workers across USA	Heathcare cost	Primary and secondary national data analysis	1. In 2007, the number of deaths due to pulmonary tuberculosis was 25 2. In 2007, the medical cost for pulmonary tuberculosis was USD0.07 billion
Lim et al. (2020) [70] / Australia	Health-care	COVID-19	2020-current	Cross-sectional study	Emergency physicians (*N* = 32)	Productivity	Hospital administration database analysis	1. 49% reduction in productivity during the COVID-19 pandemic from previously published data (*p* < 0.0001)
Matsuo et al. (2021) [71] / Japan	Health-care	COVID-19	2020-current	Cross sectional study	Tertiary hospital HCW (*N* = 660)	Employee engagement	Self-developed survey	1. 65% participants had dropout intentions
Mosteiro et al. (2020) [72] / Spain, Brazil, Portugal	Health-care	COVID-19	2020-current	Cross-sectional study	Primary health care and hospitals nurses (*N* = 659)	Presenteeism	SPS-6	1. Prevalence of presenteeism was 55, 36 and 30% for Portugese, Brazilian and Spanish participants respectively
Noorashid & Chin (2021) [73] / Brunei	Hospitality & Tourism	COVID-19	2020-current	Qualitative study	Community-based tourism owners (*N* = 16)	Business disruption, financial loss, demand shock	Semi-structured interview	1. National containment measures included lockdown and movement restrictions 2. Demand shock due to movement restriction and risk aversion 3. Participants reported disruption to local businesses, reduced earnings, and financial difficulties
Novak et al. (2021) [74] / Croatia and Serbia	Health-care	COVID-19	2020-current	Cross-sectional study	Pharmacists (*N* = 574)	Productivity	Self-developed survey	1. Containment measures included isolation (working behind acrylic glass partitions), PPE, temperature screening, provision of hand sanitizer and disinfection of work area 2. 25% participants reported negative effect on productivity due to changes in working conditions
Palmer et al. (2010) [75] / USA	Multiple	Influenza	2007–2008	Cohort study	Retail, manu-facturing and transport staff (*N* = 2013)	Absenteeism, presenteeism	Self-developed survey; items adapted from HPQ	1. The incidence of employee ILI ranged from 4.8 to 13.5% 2. Employees reporting ILI reported more absences than employees not reporting ILI (72% vs 30%, respectively; *p* < 0.001) 3. An average of 1.7 days of work absence were attributable to ILI 4. Mean ILI-related presenteeism was 2.5 hours
Richmond et al. (2020) [76] / USA	Health-care	COVID-19	2020-current	Cross-sectional study	Southeastern Surgical Congress members (*N* = 183)	Employment, productivity	Self-developed survey	1. Practices reduced staffing through paid time off (48%), furlough (40%), and termination (7%). Most participants predicted annual compensation would be moderately reduced (63.4%) 2. Participants estimated clinical productivity would be moderately reduced (48%) or extremely reduced (42%)
Schanzer et al. (2011) [77] / Canada	Multiple	Influenza/H1N1 flu	2009	Cross-sectional study	Household	Absenteeism	Statistics Canada’s Labour Force Survey data analysis	1. Hours lost due to the H1N1/09 pandemic strain more than seasonal influenza (0.2% of potential hours worked annually) 2. Estimated 0.08% of hours worked annually were lost due to seasonal influenza illnesses 3. Absenteeism rates due to influenza were estimated at 12% per year for seasonal influenza over the 1997/98 to 2008/09 seasons, and 13% for the two H1N1/09 pandemic waves 4. Employees took an average of 14 hours off due to a seasonal influenza infection, and 25 hours for the pandemic strain
Slone et al. (2021) [78] / USA	Health-care	COVID-19	2020-current	Cross-sectional study	Mental health providers (*N* = 500)	Employment	Self-developed survey	1. 22, 21, and 0.2% participants reported reduced pay, being furloughed, and laid off respectively
Tilchin et al. (2021) [79] / USA	Multiple	COVID-19	2020-current	Cross-sectional study	Amazon’s MTurk service worker (*N* = 220)	Presenteeism	Self-developed survey	1. 35% participants reported an intention to still work if they felt a little sick with COVID-19 due to financial strain
Torá-Rocamora et al. (2011) [80] / Spain	Multiple	H1N1 flu	2009	Cohort study	Catalonia workers (*N* = 3,157,979)	Absenteeism	Time series analysis of surveillance data	1. Containment measures included surveillance 2. Influenza activity peaked earlier in 2009 and yielded more cases than in previous years. Week 46 (in November 2009) had the highest number of new cases resulting in sickness absence (endemic-epidemic index 20.99; 95% CI 9.44 to 46.69)
Tsai et al. (2014) [81] / USA	Multiple	Influenza	2007–2009	Cross-sectional study	Privately insured workers (*N* = 1,860,562,007–2008, *N* = 1,953,662,008–2009)	Absenteeism	MarketScan database analysis	1. There were 2406 ILI-related work absence records in 2007–2008 and 1675 in 2008–2009 2. The mean work-loss hours per ILI were 23.6 in 2007–2008 and 23.9 in 2008–2009 3. Work-loss hours per episode were greater if the ILI episode was associated with hospitalization: 47.0 in 2007–2008 and 46.1 in 2008–2009
Turnea et al. (2020) [82] / Romania	Multiple	COVID-19	2020-current	Cross-sectional study	Company decision-makers (*N* = 203)	Demand shock, service disruption, employment, financial loss, supply chain disruption, supply cost	Self-developed survey adapted from ILO	1. Containment measures included state of emergency declaration, and quarantine of workers 2. 29% companies stopped operations 3. 81% companies reported that demand is lower than normal 4. 18% companies dismissed workers due to COVID-19 crises; 9, 2, 2, 2 and 3% dismissed 1–10%, 11–20%, 21–30%, 31–40% and over 41% workers respectively 5. 52% companies had workers on temporary leave (furlough); 4, 5, 2, 5 and 35% sent 1–10%, 11–20%, 21–30%, 31–40%, and over 41% to furlough respectively 6. 17, 27 and 44% companies reported low, medium, and high level of financial impact on business and company operations respectively 7. 9, 12, 7, 6 and 45% companies reported 10–20%, 21–30%, 31–40%, 41–50, and 51% and over average monthly revenue decrease since state of emergency established 8. 40% companies reported that raw materials are not in stock or their purchase has become very expensive
Van der Feltz-Cornelis et al. (2020) [83] / UK	Education	COVID-19	2020-current	Cross-sectional study	University staff (*N* = 1055)	Absenteeism, presenteeism	iPCQ	1. 7% participant reported sickness absenteeism 2. 26% participant experienced presenteeism
Van der Merwe et al. (2021) [84] / South Africa	Agri-culture	COVID-19	2020-current	Cross-sectional study	Wildlife ranching members (*N* = 601)	Demand shock, financial loss, employment	Self-developed survey	1. National containment measures included total lockdown 2. The estimated financial impact of COVID-19 on the private wildlife industry is USD0.99 billion 3. Average financial loss due to cancellations of hunters and ecotourist (> 77%) was USD122,100 4. The total loss in live game sales and game meat sales over the lockdown approximately USD80 million 5. 33% employees received reduced wages, 21% had to take unpaid leave, and 19% were laid off
Van Wormer et al. (2017) [85] / USA	Multiple	Influenza/H1N1 flu	2012–2016	Cross-sectional study	Marshfield workers (*N* = 1278)	Productivity	WPAI	1. There were 470 (37%) cases of influenza among workers, 179 (38%) of which are H1N1 flu 2. Influenza was significantly associated with workplace productivity loss (*P* < 0.001) 3. Regardless of vaccination, participants with A/H1N1pdm09, A/H3N2, or B infection had the greatest mean productivity loss (range, 67 to 74%), while those with non-influenza ARI had the lowest productivity loss (58 to 59%)
Webster et al. (2021) [86] / Central America	Multiple	COVID-19	2020-current	Longi-tudinal study	El Salvador, Guatemala, Honduras & Nicaragua workers (T1 *N* = 808, T2 *N* = 827)	Employment, financial loss	World Bank enterprise survey, COVID-19 survey	1. Substantial total reduction (11.7%) in employment reported by firms both at T1 and T2; huge loss of employment for the hospitality sector (T1 41%, T2 26%), whereas some sectors reported increases in employment compared to pre-pandemic (e.g. chemicals, rubber) 2. All 4 countries reported average temporary closure of > 5 weeks at T1, implying overall loss of 322,000 labour weeks. At T2, an average of further temporary closures of 2.9 weeks was reported, with an implied loss of 159,000 labour weeks 3. Workers furloughed ranged from 11 to 30% in four countries 4. 26% of firms reduced the salaries of their employees and almost a third (32%) reduced hours of work 5. Average change in sales for firms was a reduction of just under one quarter of their sales one year previously
Widodo et al. (2020) [87] / Indonesia	Transport	COVID-19	2020-current	Cross-sectional study	Engineering employees (*N* = 65)	Productivity	Self-developed survey	1. Containment measures included isolation policy, and physican distancing 2. R^2^ value (0.681) indicate that job stress and Covid-19 simultaneously affect workers’ productivity by 68%; with Covid-19 stress parameters being more influential than job stress on productivity
Yohannes et al. (2003) [88] / Australia	Multiple	Influenza	2002	Cross-sectional study	Australian workers	Absenteeism	National survei-llance system data analysis	1. National containment measures included surveillance 2. Data suggested an association between the peak in influenza activity and absenteeism 3. Influenza was responsible for 9825 hospital days in 2000–2001
Zaffina et al. (2019) [89] / Italy	Health-care	Influenza	2016–2018	Cross-sectional study	Paediatric hospital HCW (2016 *N* = 2090, 2017 *N* = 2097)	Absenteeism, work loss	Hospital record data analysis	1. The average absenteeism rate recorded a difference of 0.95 and 0.96 sickness absence days, respectively, between non-epidemic and epidemic periods 2. The total amount of days lost is 690.1 and 795.3 in 2016/2017 and 2017/2018 epidemic periods, respectively, for a total of 1.485,4 days lost 3. A total cost of USD 161,621.49 and USD 186,047.94 were calculated for 2016–2017 and 2017–2018, respectively

*ARI* acute respiratory infection, *COVID-19* Coronavirus Disease 2019, *EM* emergency medicine, *GDP* gross domestic product, *HCW* healthcare workers, *HPQ* World Health Organization Health and Work Performance Questionnaire, *H1N1* influenza A virus subtype H1N1; *ICU* intensive care unit, *ILI* influenza like illness, *ILO* International Labour Organisation, *iPCQ* iMTA Productivity Cost Questionnaire, *PPE* personal protective equipment, *SARS* Severe Acute Respiratory Syndrome, *SD* standard deviation, *SPS-6* Stanford Presenteeism Scale, *WPAI* Work productivity and activity impairment questionnaire

Most studies (47%) were assigned either a level of 3a or 3b according to the CCHMC’s Table of Evidence Levels, with 3a indicating a better-quality study than 3b, though of lower-level evidence than 1a/1b and 2a/2b. Meanwhile, several studies (36%) were assigned either a level 4a or 4b, 13% of studies either a level 2a or 2b, and 4% of studies a level 5a. According to the JBI and CEBMa tools, most studies (84%) were of good/excellent quality. The average score and range score for included studies according to the star system were as follows: (1) analytical cross-sectional studies (*n* = 22): average score 5.6, range score 3 to 8, (2) qualitative study (*n* = 6): average score 7, range score 6 to 8, (3) longitudinal studies (*n* = 5): average score 5.8, range score 3 to 7, (5) case study (*n* = 3): average score 7.3, range score 6 to 8, (6) prevalence study (*n* = 18): average score 7.3, range score 6 to 9, and (7) case control study (*n* = 1): score 6. The most frequent study design was analytical cross-sectional study (40%), followed by prevalence study (33%), qualitative study (11%), longitudinal study (9%), case study (4%), case control study (2%) and cohort study (2%). Sample sizes varied widely, ranging from 11 to 3,157,979. Of those that conducted primary studies (*n* = 34), majority (68%) utilised self-developed surveys as the mode of data collection, whereas a smaller number utilised validated tools (24%), qualitative methods (9%) and diary card (3%). Of those that performed economic analysis (*n* = 20), data analysis was performed using data retrieved from national databases (50%), hospital databases (35%), public or private insurance databases (10%), and online databases (5%). On the whole, the quality of evidence from this systematic review can be rated as good. A summary of the methodological quality of included studies is illustrated in Table 2.

**Table 2:** Quality of included studies according to CCHMC Table of Evidence Levels, JBI tools, and CEBMa tool

Author (Year)	Study Design	LOE	Q1	Q2	Q3	Q4	Q5	Q6	Q7	Q8	Q9	Q10	Q11	Overall Quality
Akazawa et al. (2003) [35]	ACS	3a	*	*	*	*	*	*	*	*	N/A	N/A	N/A	Excellent
Al-Ghunaim et al. (2021) [36]	QS	2a		*		*	*			*	*	*	N/A	Good
Alsharef et al. (2021) [37]	QS	2a		*	*	*	*			*	*	*	N/A	Good
Banerjee et al. (2021) [38]	LS	3a	N/A	N/A	*		*	N/A	*	*	*		*	Good
Bergeron et al. (2006) [39]	QS	2a		*	*		*			*	*	*	N/A	Good
Brophy et al. (2021) [40]	QS	2a	*	*	*		*			*	*	*	N/A	Good
Calvo-Bonacho et al. (2020) [41]	PS	3a	*	*	*	*	*	*	*	*		N/A	N/A	Excellent
Carroll & Smith (2020) [42]	CS	5a	*	*			*	*		*	*		N/A	Good
Challener et al. (2021) [43]	ACS	4b			*	*			*	*	N/A	N/A	N/A	Moderate
Considine et al. (2011) [44]	ACS	4a	*	*	*	*			*	*	N/A	N/A	N/A	Good
Danial et al. (2016) [45]	CS	5a	*	*	*		*	*		*	*	*	N/A	Excellent
Delaney et al. (2021) [46]	ACS	4a		*	*	*	*	*	*	*	N/A	N/A	N/A	Excellent
Duarte et al. (2017) [47]	ACS	3a		*	*	*	*	*	*	*	N/A	N/A	N/A	Excellent
Escudero et al. (2005) [48]	PS	3a	*	*	*		*	*	*	*		N/A	N/A	Good
Fargen et al. (2020) [49]	ACS	4b			*	*			*	*	N/A	N/A	N/A	Moderate
Gashi et al. (2021) [50]	PS	3a	*			*	*	*	*	*		N/A	N/A	Good
Gray et al. (2021) [51]	LS	3a	N/A	N/A	*	*	*	N/A	*	*	*	*		Excellent
Groenewold et al. (2013) [52]	PS	3a	*	*	*		*	*	*	*		N/A	N/A	Good
Groenewold et al. (2019) [53]	PS	3a	*	*	*	*	*	*	*	*		N/A	N/A	Excellent
Groenewold et al. (2020) [54]	PS	3a	*	*	*		*	*	*	*		N/A	N/A	Good
Haidari et al. (2021) [55]	ACS	4a		*	*	*	*	*	*	*	N/A	N/A	N/A	Excellent
Hammond & Cheang (1984) [56]	PS	3a	*	*	*		*	*	*	*		N/A	N/A	Good
Harrop et al. (2021) [57]	ACS	3b	*						*	*	N/A	N/A	N/A	Moderate
Hasan et al. (2021) [58]	PS	3a	*	*	*		*	*	*	*		N/A	N/A	Good
Hemmington & Neill (2021) [59]	QS	2a	*	*	*	*	*			*	*	*	N/A	Excellent
Iacus et al. (2020) [60]	PS	3a	*	*	*		*	*	*	*	*	N/A	N/A	Excellent
Jazieh et al. (2021) [61]	ACS	4a	*		*	*	*	*	*	*	N/A	N/A	N/A	Excellent
Jha et al. (2020) [62]	PS	3a	*		*		*		*	*	*	N/A	N/A	Good
Jiménez-Labaig et al. (2021) [63]	ACS	4b			*	*			*	*	N/A	N/A	N/A	Moderate
Jones et al. (2021) [64]	PS	3a	*	*	*	*	*	*	*		*	N/A	N/A	Excellent
Karatepe et al. (2021) [65]	ACS	4a			*	*	*	*	*	*	N/A	N/A	N/A	Good
Karve et al. (2013) [66]	LS	3a	N/A	N/A	*	*	*	N/A	*	*			*	Good
Keech et al. (1998) [67]	PS	3a	*	*	*	*	*	*	*	*	*	N/A	N/A	Excellent
Lee et al. (2008) [68]	PS	3a	*	*	*		*		*	*		N/A	N/A	Good
Leigh (2011) [69]	PS	3a	*	*	*	*	*	*	*	*	*	N/A	N/A	Excellent
Lim et al. (2020) [70]	ACS	4a	*	*	*	*		*	*	*	N/A	N/A	N/A	Excellent
Matsuo et al. (2021) [71]	ACS	4a		*	*	*		*	*	*	N/A	N/A	N/A	Good
Mosteiro-Diaz et al. (2020) [72]	ACS	4a	*	*	*	*	*		*	*	N/A	N/A	N/A	Excellent
Noorashid & Chin (2021) [73]	QS	2a	*	*	*	*	*			*	*	*	N/A	Excellent
Novak et al. (2021) [74]	ACS	4b	*	*	*				*		N/A	N/A	N/A	Moderate
Palmer et al. (2010) [75]	Cohort study	2a	*	*	*			*	*	*	*	*	*	Excellent
Richmond et al. (2020) [76]	ACS	4b		*		*			*	*	N/A	N/A	N/A	Moderate
Schanzer et al. (2011) [77]	PS	3a	*	*	*	*	*	*	*	*		N/A	N/A	Excellent
Slone et al. (2021) [78]	ACS	4a	*	*		*			*	*	N/A	N/A	N/A	Good
Tilchin et al. (2021) [79]	ACS	4a	*	*	*	*	*	*		*	N/A	N/A	N/A	Excellent
Torá-Rocamora et al. (2011) [80]	LS	3a	N/A	N/A	*	*	*	N/A	*	*	*		*	Excellent
Tsai et al. (2014) [81]	PS	3a	*	*	*	*	*	*	*	*		N/A	N/A	Excellent
Turnea et al. (2020) [82]	ACS	4b		*		*			*		N/A	N/A	N/A	Moderate
Van der Feltz-Cornelis et al. (2020) [83]	ACS	4a		*	*		*	*	*	*	N/A	N/A	N/A	Good
Van der Merwe et al. (2021) [84]	PS	3a	*	*	*		*		*	*		N/A	N/A	Good
Van Wormer et al. (2017) [85]	ACS	4a	*	*	*	*	*	*	*	*	N/A	N/A	N/A	Excellent
Webster et al. (2021) [86]	LS	3b	N/A	N/A	*			N/A	*	*				Moderate
Widodo et al. (2020) [87]	ACS	4b		*		*				*	N/A	N/A	N/A	Moderate
Yohannes et al. (2003) [88]	PS	3a	*	*	*		*	*	*			N/A	N/A	Good
Zaffina et al. (2019) [89]	CCS	4a		*	*	*	*			*	*		N/A	Good

*ACS* analytical cross-sectional study, *CCS* case-control study, *CS* case study, *LOE* level of evidence, *LS* longitudinal study, *PS* prevalence study, *QS* qualitative study, * star awarded, *N/A* not applicable

(1) The CCHMC Table of Evidence classifies level of evidence for individual studies by study design, domain, and quality, with level 1 representing the highest level and indicating the strongest evidence, and level 5 representing the lowest level and indicating the weakest evidence. In addition, the Joanna Briggs Institute (JBI) tools and Center of Evidence Based Management (CEBMa) case study critical appraisal checklist were used to further subclassify studies at each level to either “a” or “b”, which signifies good quality and lesser quality study respectively in terms of methodological quality

(2) Some questions are indicated as N/A because the quality tool for that specific study design has a certain number of quality appraisal checklist, e.g., JBI for ACS has 8 quality appraisal checklists, and Q9 to Q11 do not apply

### Socioeconomic impacts of airborne and droplet-borne infectious diseases in industries

**Fig. 2:**
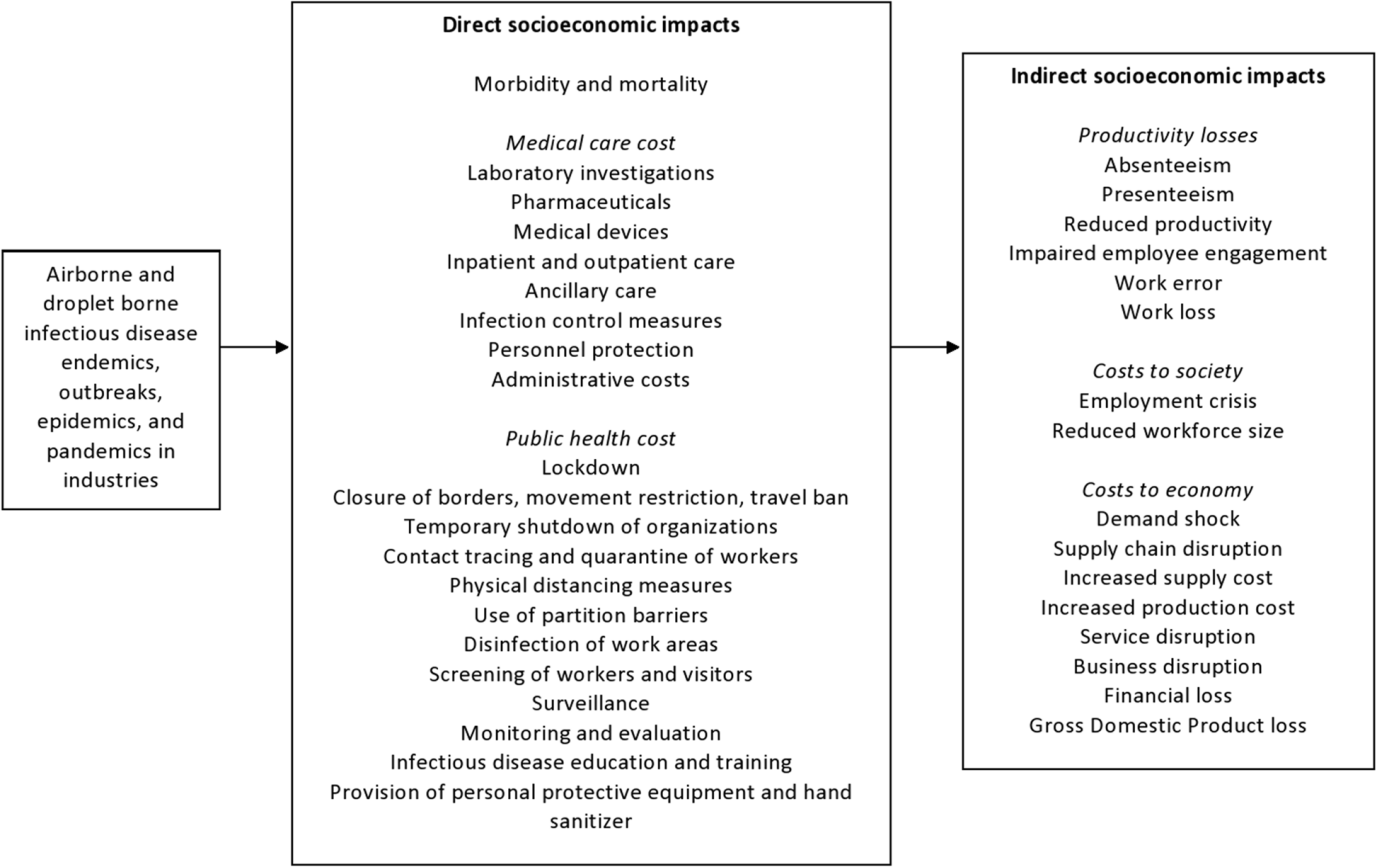
Framework for socioeconomic impact of airborne and droplet-borne infectious disease on industries based on empirical findings

A variety of socioeconomic impacts were reported by studies included in this review, as outlined in Fig. 2. They include direct impacts, i.e. repercussions occurring during the hazard event, as well as indirect impacts, i.e. subsequent changes given the direct impact [90].

- Direct impacts of airborne and droplet-borne infectious diseases in industries

Direct socioeconomic impacts such as morbidity and mortality and its associated healthcare costs due to infectious diseases outbreaks were reported by included studies. Exposure to influenza, influenza A (H1N1), SARS-CoV, and SARS-CoV-2 viruses, *Mycobacterium tuberculosis*, and norovirus had resulted in influenza like illness (ILI), febrile illness, pneumonia, COVID-19 infection, pulmonary tuberculosis, and gastroenteritis among workers [45, 48, 49, 61, 63, 69, 75, 85]. Moreover, a small percentage of those who developed pneumonia from exposure to SARS-CoV virus and *Mycobacterium tuberculosis* had succumbed to death [48, 69].

In addition, substantial healthcare costs were reported because of these outbreaks. During influenza epidemics, the average per-patient influenza-related medical cost ranged from USD239 to USD301, whereas the total healthcare expenditure for workers of a United Kingdom (UK) pharmaceutical company amounted to USD2,512.16, due to ILI-related medical, inpatient, outpatient, general practitioner/physician office, emergency department, pharmacy, and ancillary care utilization and costs [66, 67]. Meanwhile, the average medical costs due to hospital, professional services, pharmaceuticals, medical devices, and nursing homes for workers with pulmonary tuberculosis was reported to amount to USD0.07 billion in 2017 [69]. Included studies had also highlighted public health costs taken to contain infectious diseases outbreaks such as lockdown, closure of borders, restriction of free movement, travel ban, temporary shutdown of organisations, screening of workers and visitors, quarantining of workers, physical distancing measures, use of partition barriers, infection control and disinfection of work areas, infectious disease-related training, provision of personal protective equipment and hand sanitizers, and surveillance [37, 39, 40, 48, 50, 59, 60, 73, 74, 80, 82, 84, 87, 88]. To control the three-month norovirus outbreak in a Scottish hospital, the healthcare costs included cleaning costs (USD5,021.52), incident management team (USD64,562.41), and laboratories (USD2,295.55) [45].

b) Indirect impacts of airborne and droplet-borne infectious diseases in industries

Following the impacts above, indirect socioeconomic impacts of infectious diseases outbreaks including productivity losses, costs to society, and costs to economy were also reported. Absenteeism was observed among workers across multiple industries during influenza, H1N1 flu, SARS, and COVID-19 outbreaks. Workers who were exposed to influenza had 1.3 to 2.8 workdays missed and 14.0 to 23.9 work hours lost per ILI [35, 43, 53, 67, 75, 77, 81], and there was a 800% increase in absenteeism rate during epidemics compared to non-epidemic periods [47, 56, 88, 89]. Compared to seasonal influenza, hours lost due to the H1N1 pandemic strain were higher (0.2% of potential hours worked annually) [77], and workers with H1N1 flu had 3.73 workdays missed and 25 hours work hours lost [44, 52, 77, 80]. Meanwhile, exposure to SARS had resulted in 1.4 missed workdays per 100 staff-days observed [48]. Finally, an average of 4.9 cases of sickness leave per 1000 workers were observed during the COVID-19 pandemic, which represented a dramatic increase compared to previous years (4.9 cases vs 2.5 cases per 1000 workers in March 2020 vs. 2017, 2018, and 2019) [41, 54, 65, 83]. All activity sectors were impacted, with the highest rate of absenteeism observed among workers in the healthcare, services, production, and transportation industries [41, 54].

Concurrently, presenteeism among workers was also reported during influenza, H1N1 and COVID-19 outbreaks [43, 67, 72, 79, 83]. During the influenza/H1N1 flu epidemic, 73% workers reported that the illness had interfered with work, 81% workers who had returned to work while symptomatic felt only moderately effective, and a mean productivity loss ranging from 67 to 74% was reported [67, 85]. This culminated in workers with ILI being less productive for 4.8 hours each day worked while ill (2.5 hours each day with ILI symptoms) [75]. Meanwhile, workers across industries reported being less productive and efficient at work during the COVID-19 pandemic, which amounted to a 49% reduction in productivity from previously published data (*p* < 0.0001) [36–38, 46, 55, 57, 61, 70, 74, 76, 87]. For healthcare workers in particular, in addition to productivity losses during the pandemic, impaired work quality and reduced employee engagement were also observed [36], as 12% reported increased medical errors [55], 23% had doubts about their medical vocation [63], and 21 to 65% had moderate or very serious consideration about leaving the workforce [46, 62, 71].

Correspondingly, increased costs to industries in the form of work loss were observed during infectious diseases outbreaks. In the USA, the total salary paid out for sickness absenteeism in the two-week period of peak absenteeism during the epidemic were much greater compared to non-epidemic periods (USD60,776 vs. USD36,290) [56], and the average work loss and influenza-related productivity loss were valued at USD137 per person [35] and USD42,581 per 100,000 health plan member [66] respectively. In the UK, the overall total cost of missed workdays for ILI among workers of a large UK pharmaceutical company was valued at USD159,769.67 [67]. Meanwhile, work loss due to exposure to influenza resulted in a total cost of USD161,621.49 and USD186,047.94 for 2016–2017 and 2017–2018 respectively in Italy [89], and led to USD152.12 average productivity loss per person per year in Hong Kong [68]. On the other hand, the total cost of staff absence due to norovirus exposure was estimated to be USD16,232.42 [45]. Finally, the increased sick leave during COVID-19 pandemic had translated into USD4374.81 per 100 affiliated workers across industries [41].

In terms of costs to society, employment crises and reduced workforce size were reported by included studies, especially in the wake of COVID-19 pandemic. Studies reported workers across industries being terminated (0.2–41%), furloughed (6–56%), or made to go on paid time off (48%) during the COVID-19 pandemic [49, 50, 58, 59, 62, 64, 76, 78, 82, 84, 86]. During this period, companies across multiple industries had also reduced either the salaries (17–33%) or hours of work (32–68%) of their employees [62, 64, 78, 84, 86]. Correspondingly, studies had also reported reduced workforce size and staff shortages (48%) during the COVID-19 pandemic [37, 40, 51], which was similarly apparent during the SARS [39] and H1N1 flu epidemics [47]. A small number of industrial sectors (e.g. chemical, plastics and rubber industry) had however showed increases in employment during the COVID-19 pandemic compared to pre-pandemic periods [86]. In the aviation industry alone, job losses in the aviation industry had been forecasted to reach 25 to 30 million at the end of 2020 [60].

Costs to economy was also extensively reported by included studies, especially as a result of the COVID-19 pandemic. Studies conducted in multiple industries [82], including the transport [60], hospitality and tourism [59, 73], and agriculture industries [58, 84] reported demand shock during the COVID-19 pandemic due to movement restrictions, risk aversion, and lower consumerism. The exception to this is the study conducted in the construction industry, which had reported that there was increased demand for home improvement and renovation products and supplies from local supplier and manufacturers [37]. Disruptions to supply chain, services, as well as businesses were also observed during the COVID-19 pandemic. Studies conducted across multiple industries described material shortages and delays in material delivery, which caused significant schedule disruptions [37, 50, 82] as well as cessation of operations during this time [62, 82]. This was similarly reported during the SARS outbreak, where healthcare workers had reported program stoppages [39]. Besides that, increased supply and production costs were also noted since the onset of COVID-19 pandemic. In the healthcare industry, an increase of USD107,040 to USD535,198 in supply costs were reported [42]. Similarly, the total production costs (primary fixed costs, operation costs, feed costs, medicinal costs) in the agriculture industry had also increased [58] and 40% companies across industries reported that raw materials were not in stock or their purchase has become very expensive [82].

The COVID-19 pandemic had also reportedly led to companies suffering financial losses. In the healthcare industry, the reduction in surgical and clinic volume as well as substitution of acute care for critical care in a Washington hospital were estimated to result in revenue loss amounting to USD13 to 117 million per year [42]. In the agriculture industry, the estimated financial loss incurred due to cancellations of hunters and ecotourist as well as loss in live game sales and game meat sales over lockdown in South Africa were reported to amount to USD0.99 billion loss to the private wildlife industry, whereas finfish farmers across Dhobaura, Bangladesh described receiving less profits and suffering a real price reduction of USD0.16/kg [58, 84]. In the hospitality and tourism industry, café income had decreased in New Zealand and tourism owners in Brunei reported reduced earnings and financial difficulties, which had led to companies with low margins and poor cashflow going out of business [59, 73]. Meanwhile, across multiple industries in Central America, Romania, and Kosovo, firms observed 25% reduction in sales compared to the year previously [86], reduced average revenue since state of emergency was established [82], and losses of USD32,643.53, USD316,624.61, USD804,205.05, and USD864,353.31 for microenterprises, small enterprises, medium enterprises, and large companies respectively [50]. In the transport industry alone, GDP loss in the transport industry were forecasted to range from 1.41 to 1.67% globally by the end of 2020 [60].

## Discussion

The primary aim of this systematic review was to determine the socioeconomic impacts of airborne and droplet-borne infectious diseases on industries. The findings of 55 studies encompassing multiple industries across the globe indicate that significant direct and indirect socioeconomic costs were incurred as a result of COVID-19, influenza, influenza A (H1N1), SARS, tuberculosis and norovirus outbreaks, as highlighted in Fig. 2. According to the framework derived from empirical findings, outbreaks of airborne and droplet-borne infectious diseases in industries cause illnesses, deaths, high medical and public health costs, which in turn lead to significant productivity, social, and economic costs. These observations are in line with the model published by Phua (2005) [91], in which the most apparent costs following infectious diseases outbreaks include morbidity, mortality and direct costs of medical care and public health interventions, as well as indirect costs attributable to the loss of productivity resulting from morbidity, mortality, and related health interventions. Following the methodological assessment of included studies according to the JBI and CEBMa tools, the quality of evidence from this systematic review can be rated as good. Thus, the findings from this systematic review provide reasonably robust evidence of the socioeconomic impacts of airborne and droplet-borne diseases on industries.

As shown in this systematic review, airborne and droplet-borne infectious diseases were significant causes of morbidity and mortality among workers, which ranged from self-limiting ILI from influenza infection to pneumonia from SARS infection to death from pulmonary tuberculosis. Concurrently, substantial costs incurred from the use of healthcare resources including healthcare expenditures for the diagnosis and treatment of workers, as well as public health preventive and control measures for managing the diseases at workplaces and communities. Indeed, influenza epidemics had accounted for USD1–3 billion, USD1.1 billion, USD300 million, and USD7.90 million in direct medical costs in USA, Germany, France and South Korea respectively [8, 92]. Meanwhile, the direct medical costs due to 2009 H1N1 pandemic were estimated at USD291.7 million, 37 times the costs compared to seasonal influenza [92], whereas the COVID-19 pandemic had led to a total direct medical cost of USD163.4 billion in the USA alone [93]. On the other hand, direct medical costs attributable to tuberculosis, an endemic disease, was USD0.07 billion [69]. In this regard, the morbidity and mortality of infectious diseases and associated health costs varied widely, and is dependent on multiple factors. These factors include the transmissibility, virulence, and case fatality rate of the pathogen, viral variants, national demography, prevalence of comorbidities, as well as the scale of the outbreak, public health capacity and response, and availability of treatment [94].

In addition to the direct costs of infectious diseases outbreaks, the indirect costs has been shown to be 5 to 10-fold greater than direct costs and stems largely from losses in work productivity [8]. In this study, the average workdays missed due to exposure to airborne and droplet-borne infectious diseases ranged from 1.3 to 3.73. This may be attributable not only to workers getting ill but also to risk aversion behaviours adopted by workers to prevent becoming infected [54]. Moreover, for large-scale infectious diseases outbreaks, sickness absence from school as well as closure of schools may lead to parents having to take time off work to care for their children [5, 8]. Concurrently, presenteeism, which had resulted in 49 to 74% reduction in productivity, was also reported in this study. This may be due to various factors, including professional obligation, “lack of cover”, job insecurity, high job demand, inflexible work condition, peer pressure, and presenteeism culture [95]. According to Smith et al. (1993) [96], even mild influenza had resulted in a reduction of reaction times by 20 to 40%, which may contribute towards impaired work performance with adverse effects on health and safety at work (e.g. medical errors) as observed in this study. Furthermore, studies suggest that the increased tendency of workers to remain indoors due to public health measures instituted during infectious diseases outbreaks may also adversely impact health and lead to poorer work performance, due to increased exposure to indoor air pollutants [97].

Due to the outbreak scale of the 2009 H1N1 pandemic and COVID-19 pandemic, national policies such as lockdowns, movement restrictions, and restricting industries sector operation to only those considered essential services had to be undertaken in efforts to control the pandemic [92]. These measures, coupled with risk aversion among the general public, had led to supply shock due to temporary closure of businesses deemed non-essential, as well as demand shock due to decreased consumption and travel among the general public [98]. Due to the above, hundreds of millions of workers found themselves losing work, both in formal and informal labour markets [99]. As demonstrated in this study, workers across industries had reported being terminated, furloughed, made to go on paid leave, or having their wages or hours of work reduced during infectious diseases outbreaks. In the USA alone, nearly 6.6 million workers filed for unemployment benefits by the end of March 2020 due to COVID-19, disrupting a decade-long streak of growth in employment [98]. In this aspect, industries with high proportions of temporary jobs, inflexible working arrangements, and reliance on migrant workforces experienced greater labour losses [6, 100, 101]. As an aftermath of infectious diseases outbreaks, the employment crises may lead to more systemic long-term effect changes, including multiplier effects on employment, household income, and food security [6].

In terms of infectious diseases’ costs to economy, the health services, transport, hospitality and tourism industries were affected the most [5, 6, 102]. During the COVID-19 pandemic, countries’ health systems had been partly or entirely interrupted [102]. High numbers of active cases had overwhelmed the health delivery system and its capacity to maintain other essential health services [103]. Moreover, frontline healthcare providers were getting infected at a greater rate compared to the general public and the quarantine measures to control the spread of infectious diseases had resulted in shortage in healthcare staffing, further stressing the health system [6, 101]. Meanwhile, border closure, travel ban, suspension of flight operations globally, restrictions on public gatherings, as well as contagion fears had inhibited social and recreational activities and reduced spending activities, negatively impacting the transport, tourism and hospitality industries [4, 101, 102]. During the 2003 SARS outbreak, Asia-Pacific carriers and North American carriers saw USD6 billion and USD1 billion loss in revenue respectively [104], whereas H1N1 influenza led to USD2.8 billion loss in revenue for Mexico’s tourism industry [105]. The 2015 MERS outbreak in South Korea and Saudi Arabia had led to USD10 billion and USD5 billion loss in revenue respectively for the tourism industry [4, 106]. On a larger scale, the COVID-19 pandemic had led to an immediate collapse in demand in the global tourism and leisure industry, 50 million job loss globally, and USD2.86 trillion loss in revenue due to significant slumps in domestic and international tourism [5, 6, 107].

Closure of borders, reduced personal spending and demand for goods, and halts in non-essential imports during infectious diseases outbreaks had also led to demand shocks across multiple industries [6]. The 2015 MERS outbreak had resulted in 10, 8.6, 6.3, 2.4, 1.6, and 0.9% drop in production for the accommodation and food, entertainment and recreation, publishing, communication, and information, transportation and storage, wholesale and retail, and electricity and air conditioning sectors respectively [106]. During the COVID-19 pandemic, government-imposed shutdown had led to the temporary closure of major manufacturing companies across the globe, causing global supply chain disruptions for raw materials and intermediate products as well as disruptions in international and regional trade [5, 98, 108], which had led to material shortages, increased supply and production costs, as well as service disruptions as observed in this study. Indeed, entire systems including production, transportation, marketing, distribution and consumption had been adversely impacted, leading to reduced profit margins and financial strain on businesses [6, 98]. MSME, especially those reliant on intermediate goods imported from affected regions, faced greater difficulty in enduring the disruption [98]. Indeed, according to previous studies, almost all MSME in South Asia were unable to sustain themselves through lockdown and were forced to close their operations during the COVID-19 pandemic [6].

Other industries were not spared from infectious diseases outbreaks, as impacts on industries have knock-on effects on one another due to their interdependencies [98]. Indeed, as an aftermath to the 2003 SARS outbreak, restrictions and cancellation in the transport industry had impacted multinational industries such as oil, for which demand had reduced by 300,000 barrels a day in Asia [104]. During the COVID-19 pandemic, all sectors of the world economy had been affected [5], and in fact, it became a global systemic economic risk due to the high globalization and interconnectedness among the different industries and sectors of the economy [6, 101]. Nevertheless, a small number of industries had performed better during pandemic, reflecting changes in consumer spending and market behaviour [101]. For example, South Korea market chain stores reported increased online sales [4], and the food sector, including distribution and retailing, experienced huge demands on food products due to panic-buying and stockpiling of food among the general public [5]. Similarly, stay-at-home orders had contributed to the increased demand for home improvement and renovation products in the construction industry, as observed in this study.

Overall, the total global economic loss due to influenza, H1N1, SARS, and COVID-19 epidemics were estimated to reach USD600 billion, USD360 billion, USD40 billion, and USD8.5 trillion respectively [109–112]. According to the World Bank, the global economy was forecasted to shrink by 5.2% by the end of 2020 due to COVID-19, the worst recession since World War II [113]. In this regard, the economic impacts on poorer countries is higher due to already strained economic conditions and reduced health capacity to cope with pandemic shocks [6]. This was reflected in the findings of this study, in which COVID-19 financial impacts did not spare even larger enterprises across multiple industries in Kosovo, a middle-income economy. Similarly, the 2014–2015 Ebola outbreaks in Liberia, a low-income economy, had overwhelmed the economy due to the rise in public health expenditure, economic collapse, and revenue decline [7]. In addition, the disparity in economic downfall between countries may also be attributed to vastly different sociocultural and politico-economic circumstances. For example, the devastation of COVID-19 on Pakistan, a middle-income economy, have been suggested to be due to distinguishable sociocultural patterns such as lower observance of preventative measures due to natives’ fatalistic religious beliefs, communal living practices, cultural norms that promote disease transmission such as handshaking and hugging, as well as food scarcity, low economic resources, and poor and corrupt governance [114].

The extent of the socioeconomic impacts of airborne and droplet-borne infectious diseases on industries will depend on the several factors. Firstly, the scale and protractedness of the outbreak will determine the necessary global and domestic actions and policy measures to contain the outbreak and the ensuing immediate and long-term economic costs [4, 115]. In this regard, endemic infectious diseases such as tuberculosis may inflict substantial but steady disease burden and associated healthcare costs, whereas epidemic infectious diseases such as influenza may quickly overwhelm the health system and necessitate public health measures that disrupts economic and other socially valuable activity [116]. Secondly, the preparedness of health systems to manage and control the outbreak, as well as the availability of effective vaccines and enhanced diagnostic tests, will also influence the resulting economic shocks [4]. Indeed, in any major outbreaks, striking the balance between public health gains and economic costs of containing the disease often proves to be politically difficult [117]. In these scenario, the socioeconomic impacts of infectious diseases may be mitigated by economic support deployed by governmental and developmental agencies during the pandemic. For example, Europe had pledged a €1.7 trillion rescue package in an effort to dampen the economic repercussions of COVID-19 on European countries [5].

This study is not without limitations. Firstly, socioeconomic data collected may become dated even before they are released or published due to the global pandemic continually developing and advancing at exceptionally rapid pace [117]. Thus, the findings of this study may have changed since August 2021. Nevertheless, our study findings may provide a perspective of the recent past that may be utilised by policy makers, public health practitioners, and other stakeholders. Moreover, as research related to the pandemic is continuing, we do not have the complete understanding of the socioeconomic impacts of COVID-19. This is especially as at present, the trajectory of SARS-CoV-2 virus and its impacts within any given country remains uncertain and is difficult to predict reliably [117]. Thirdly, in-depth quantitative analysis was not possible due to the heterogeneity of the studies included, and direct and indirect cost values were provided when and if available. Indeed, this limitation has been reported by previous studies, which described quantitative impact data being constrained by differing methodologies that result in estimates that were not comparable across and even within countries [4]. Finally, a drawback of the systematic review’s broad approach is that a wide range of outcomes were observed from countries with vastly different cultural and economic circumstances, which may not transfer easily to a specific industry or country.

Equally, it is important to note the strengths of this systematic review. This systematic review was able to elicit valuable findings in relation to the full extent and range of socioeconomic impacts of airborne and droplet-borne infectious diseases on multi-sectoral industries, which have not been attempted previously. Furthermore, measures were taken to ensure the robustness and quality of this systematic review, by conducting it in accordance to the PRISMA guidelines, searching through multiple large databases, using comprehensive and exhaustive search terms, and assessing the methodological quality of included studies using established quality assessment tools.

## Conclusion

From this systematic review, it is evident that airborne and droplet-borne infectious diseases have the potential to inflict severe socioeconomic costs on regional and global industries and sectors. In this aspect, bold policy measures and innovative mechanism are warranted to sustain economic growth and financial stability during infectious diseases outbreaks, especially those reaching pandemic levels. To this end, strengthening disease surveillance, prevention, preparedness, and response systems, as well as investments in vaccine development and distribution need to be prioritized to safeguard against the threat of infectious diseases. In addition, public health policies such as coordinated and consistent stay-at-home orders across multiple jurisdictions, rapid scale-up of testing, and rapid and accurate communication of mitigation plans to the public via social media forums have been advocated as measures to control pandemics [118]. Meanwhile, digital health innovations such as the use of telehealth, web-based tools, and mobile applications for healthcare delivery, public health informatics, and public education, and the utilisation of computer programmes such as the Geographic Information System (GIS) software, trackers, and prediction models for surveillance and risk mapping, are examples of inventive measures that could be employed during infectious diseases outbreaks [119]. Further research is needed to better understand infectious diseases’ long-term socioeconomic impacts to support improved industry preparedness and response capacity for ongoing and future outbreaks. To ensure informed systems and sector-specific cost-sharing strategies for optimal global health and economic security, public and private stakeholders at local, national, and international levels must ultimately work together.

### Supplementary Information

**Additional file 1.**

**Additional file 2.**

## Data Availability

All data generated or analysed during this study are included in this published article and its supplementary information files.

## References

[1] The World Bank. World Bank Open Data. 2022. Available from: https://data.worldbank.org. Accessed 9 Jan 2023.

[2] Pamuk S, van Zanden JL, Broadberry S, O’Rourke KH (2010). Chapter 9: standard of living. The Cambridge economic history of modern Europe: Volume 1.

[3] Costa D, Steckel RH, Steckel RH, Floud R (1997). Long-term trends in health, welfare, and economic growth in the United States. Health and welfare during industrialization.

[4] Smith KM, Machalaba CC, Seifman R, Feferholtz Y, Karesh WB (2019). Infectious disease and economics: the case for considering multi-sectoral impacts. One Health.

[5] Nicola M, Alsafi Z, Sohrabi C, Kerwan A, Al-Jabir A, Iosifidis C (2020). The socio-economic implications of the coronaviirus pandemic (COVID-19): a review. Int J Surg..

[6] Rasul G, Nepal AK, Hussain A, Maharjan A, Joshi S, Lama A (2021). Socio-economic implications of COVID-19 pandemic in South Asia: emerging risks and growing challenges. Front Sociol..

[7] Shang Y, Li H, Zhang R (2021). Effects of pandemic outbreak on economies: evidence from business history context. Front Public Health.

[8] Szucs T (1999). The socio-economic burden of influenza. J Antimicrob Chemother..

[9] Centers for Disease Control and Prevention (2023). Norovirus burden and trends United States of America.

[10] Silva S, Arinaminpathy N, Atun R, Goosby E, Reid M (2021). Economic impact of tuberculosis mortality in 120 countries and the cost of not achieving the sustainable development goals tuberculosis targets: a full-income analysis. Lancet Glob Health.

[11] Wang CC, Prather KA, Sznitman J, Jimenez JL, Lakdawala SS, Tufekci Z (2021). Airborne transmission of respiratory viruses. Science..

[12] Larson E (1997). Social and economic impact of infectious diseases -- United States. Clin Perfor. Qual Healthc..

[13] Roberts JA, Cumberland P, Sockett PN, Wheeler J, Rodrigues LC, Sethi D (2003). The study of infectious intestinal disease in England: socio-economic impact. Epidemiol Infect..

[14] Bashir MF, Ma B, Shahzad L (2020). A brief review of socio-economic and environmental impact of COVID-19. Air Qual Atmos Health.

[15] Lenzen M, Li M, Malik A, Pomponi F, Sun YY, Wiedmann T (2020). Global socio-economic losses and environmental gains from the coronavirus pandemic. PLoS One.

[16] Klepser ME (2014). Socioeconomic impact of seasonal (epidemic) influenza and the role of over-the-counter medicines. Drugs..

[17] Shapovalova NDF, Donadel M, Jit M, Hutubessy R (2015). A systematic review of the social and economic burden of influenza in low- and middle-income countries. Vaccine..

[18] Ceylan RF, Ozkan B (2020). The economic effects of epidemics: from SARS and MERS to COVID-19. Res J Adv Humanit..

[19] Tanaka S (2022). Economic impacts of SARS/MERS/COVID-19 in Asian countries. Asian Econ Policy Rev..

[20] Moher D, Liberati A, Tetzlaff J, Altman DG. Preferred reporting items for systematic reviews and Meta-analyses: the PRISMA statement. Ann Intern Med. 2009;151(4). 10.7326/0003-4819-151-4-200908180-00135 19622511

[21] Tortorella G, Narayanamurthy G, Filho MG, Staudacher AP, Mac Cawley AF (2021). Pandemic's effect on the relationship between lean implementation and service performance. J Serv Theory Pract..

[22] Narayanamurthy G, Tortorella G (2021). Impact of COVID-19 outbreak on employee performance – moderating role of industry 4.0 base technologies. Int J Prod Econ..

[23] Novitasari D, Sasono I, Asbari M (2020). Work-family conflict and worker’s performance during COVID-19 pandemic: what is the role of readiness to change mentality?. Int J Sci Manag Stud..

[24] Giorgi G, Lecca LI, Alessio F, Finstad GL, Bondanini G, Lulli LG (2020). COVID-19-related mental health effects in the workplace: a narrative review. Int J Environ Res Public Health..

[25] Ather B, Mirza TM, Edemekong PF (2021). Airborne precautions.

[26] World Health Organization (2020). Modes of transmission of virus causing COVID-19: implications for IPC precaution recommendations.

[27] Lin LL. The socioeconomic impacts of COVID-19 in Malaysia: Policy review and guidance for protecting the most vulnerable and supporting enterprises. 2020. Available from: https://www.ilo.org/asia/publications/WCMS_751600/lang-en/index.htm. Accessed 9 Jan 2023.

[28] Fleiss JL, Levin B, Paik MC (1981). Statistical methods for rates and proportions.

[29] Cincinnati Children's Hospital Medical Center (2012). Table of evidence levels: levels of individual studies by domain, study design, & quality.

[30] Joanna Briggs Institute (2022). Critical appraisal tools the University of Adelaide.

[31] Center of Evidence Based Management. Critical appraisal checklist for a case study. 2014. Available from: https://www.cebma.org. Accessed 9 Jan 2023.

[32] Ma LL, Wang YY, Yang ZH, Huang D, Weng H, Zeng XT (2020). Methodological quality (risk of bias) assessment tools for primary and secondary medical studies: what are they and which is better?. Mil Med Res..

[33] Hannes K, Lockwood C, Pearson A (2010). A comparative analysis of three online appraisal instruments' ability to assess validity in qualitative research. Qual Health Res..

[34] The Campbell and Cochrane Economics Methods Group (CCEMG), Evidence for Policy and Practice Information and Coordinating Centre (EPPI-Centre). CCEMG – EPPI-Centre cost converter v.1.6. 2019. Available from: https://eppi.ioe.ac.uk/costconversion/. Accessed 25 July 2023.

[35] Akazawa M, Sindelar JL, Paltiel AD (2003). Economic costs of influenza-related work absenteeism. Value Health.

[36] Al-Ghunaim TA, Johnson J, Biyani CS, O'Connor D (2021). Psychological and occupational impact of the COVID-19 pandemic on UK surgeons: a qualitative investigation. BMJ Open.

[37] Alsharef A, Banerjee S, Uddin SMJ, Albert A, Jaselskis E (2021). Early impacts of the COVID-19 pandemic on the United States construction industry. Int J Environ Res Public Health.

[38] Banerjee S, Lim KHJ, Murali K, Kamposioras K, Punie K, Oing C (2021). The impact of COVID-19 on oncology professionals: results of the ESMO resilience task force survey collaboration. ESMO Open..

[39] Bergeron SM, Cameron S, Armstrong-Stassen M, Pare K (2006). Diverse implications of a national health crisis: a qualitative exploration of community nurses' SARS experiences. Can J Nurs Res..

[40] Brophy JT, Keith MM, Hurley M, McArthur JE (2021). Sacrificed: Ontario healthcare Workers in the Time of COVID-19. New Solut..

[41] Calvo-Bonacho E, Catalina-Romero C, Fernández-Labandera C, Fernández-Meseguer A, González-Quintela A, Martínez-Muñoz P (2020). COVID-19 and sick leave: an analysis of the Ibermutua cohort of over 1,651,305 Spanish Workers in the First Trimester of 2020. Front Public Health.

[42] Carroll NW, Smith DG (2020). Financial implications of the CoviD-19 epidemic for hospitals: a case study. J. Health Care Finance.

[43] Challener DW, Breeher LE, Frain J, Swift MD, Tosh PK, O'Horo J (2021). Healthcare personnel absenteeism, presenteeism, and staffing challenges during epidemics. Infect Control Hosp Epidemiol..

[44] Considine J, Shaban RZ, Patrick J, Holzhauser K, Aitken P, Clark M (2011). Pandemic (H1N1) 2009 influenza in Australia: absenteeism and redeployment of emergency medicine and nursing staff. EMA - Emerg Med Australas..

[45] Danial J, Ballard-Smith S, Horsburgh C, Crombie C, Ovens A, Templeton KE (2016). Lessons learned from a prolonged and costly norovirus outbreak at a Scottish medicine of the elderly hospital: case study. J Hosp Infect..

[46] Delaney RK, Locke A, Pershing ML, Geist C, Clouse E, Precourt Debbink M, et al. Experiences of a health System's faculty, staff, and Trainees' career development, work culture, and childcare needs during the COVID-19 pandemic. JAMA Netw Open. 2021;4(4). 10.1001/jamanetworkopen.2021.3997 PMC8019096 33797552

[47] Duarte F, Kadiyala S, Masters SH, Powell D (2017). The effect of the 2009 influenza pandemic on absence from work. Health Econ..

[48] Escudero IHG, Chen MI, Leo YS (2005). Surveillance of severe acute respiratory syndrome (SARS) in the post-outbreak period. Singap Med J..

[49] Fargen KM, Leslie-Mazwi TM, Klucznik RP, Wolfe SQ, Brown P, Ansari SA (2020). The professional and personal impact of the coronavirus pandemic on US neurointerventional practices: a nationwide survey. J NeuroInterv Surg..

[50] Gashi A, Sopa I, Havolli Y (2021). The impact of COVID-19 on economic aspects of business enterprises: the CASE of Kosovo. Manag-J Contemp Manag Issues.

[51] Gray BM, Vandergrift JL, Barnhart BJ, Reddy SG, Chesluk BJ, Stevens JS (2021). Changes in Stress and Workplace Shortages Reported by U.S. Critical Care Physicians Treating Coronavirus Disease 2019 Patients∗. Crit Care Med..

[52] Groenewold MR, Konicki DL, Luckhaupt SE, Gomaa A, Koonin LM (2013). Exploring national surveillance for health-related workplace absenteeism: lessons learned from the 2009 influenza a pandemic. Disaster Med Public Health Prep..

[53] Groenewold MR, Burrer SL, Ahmed F, Uzicanin A, Luckhaupt SE (2019). Health-related workplace absenteeism among full-time workers—United States, 2017–18 influenza season. Morb Mortal Wkly Rep..

[54] Groenewold MR, Burrer SL, Ahmed F, Uzicanin A, Free H, Luckhaupt SE (2020). Increases in health-related workplace absenteeism among Workers in Essential Critical Infrastructure Occupations during the COVID-19 pandemic - United States, march-April 2020. MMWR Morb Mortal Wkly Rep..

[55] Haidari E, Main EK, Cui X, Cape V, Tawfik DS, Adair KC (2021). Maternal and neonatal health care worker well-being and patient safety climate amid the COVID-19 pandemic. J Perinatol..

[56] Hammond GW, Cheang M (1984). Absenteeism among hospital staff during an influenza epidemic: implications for immunoprophylaxis. Can Med Assoc J..

[57] Harrop C, Bal V, Carpenter K, Halladay A (2021). A lost generation? The impact of the COVID-19 pandemic on early career ASD researchers. Autism Res..

[58] Hasan NA, Heal RD, Bashar A, Bablee AL, Haque MM. Impacts of COVID-19 on the finfish aquaculture industry of Bangladesh: a case study. Mar Policy. 2021;130.

[59] Hemmington N, Neill L. Hospitality business longevity under COVID-19: the impact of COVID-19 on New Zealand’s hospitality industry. Tour Hosp Res. 2021;22.

[60] Iacus SM, Natale F, Santamaria C, Spyratos S, Vespe M. Estimating and projecting air passenger traffic during the COVID-19 coronavirus outbreak and its socio-economic impact. Saf Sci. 2020;129. 10.1016/j.ssci.2020.104791 PMC7200368 32377034

[61] Jazieh AR, Coutinho AK, Bensalem AA, Alsharm AA, Errihani H, Mula-Hussain L (2021). Impact of the COVID-19 pandemic on oncologists: results of an international study. JCO Glob Oncol.

[62] Jha S, Shah S, Calderon MD, Soin A, Manchikanti L (2020). The effect of covid-19 on interventional pain management practices: a physician burnout survey. Pain Physician.

[63] Jiménez-Labaig P, Pacheco-Barcia V, Cebrià A, Gálvez F, Obispo B, Páez D (2021). Identifying and preventing burnout in young oncologists, an overwhelming challenge in the COVID-19 era: a study of the Spanish Society of Medical Oncology (SEOM). ESMO Open.

[64] Jones AM, Clark JS, Mohammad RA (2021). Burnout and secondary traumatic stress in health-system pharmacists during the COVID-19 pandemic. Am J Health-Syst Pharm : AJHP : Off J Am Soc Health-Syst Pharm..

[65] Karatepe OM, Saydam MB, Okumus F (2021). COVID-19, mental health problems, and their detrimental effects on hotel employees’ propensity to be late for work, absenteeism, and life satisfaction. Curr Issue Tour..

[66] Karve S, Meier G, Davis KL, Misurski DA, Wang CC (2013). Influenza-related health care utilization and productivity losses during seasons with and without a match between the seasonal and vaccine virus B lineage. Vaccine..

[67] Keech M, Scott A, Ryan P (1998). The impact of influenza and influenza-like illness on productivity and healthcare resource utilization in a working population. Occup Med..

[68] Lee KK, Li SC, Kwong KS, Chan TY, Lee VW, Lau JT (2008). A study of the health and economic effects of influenza-like illness on the working population under different working environments of a large corporation in Hong Kong. J Med Econ..

[69] Leigh JP (2011). Economic burden of occupational injury and illness in the United States. Milbank Q..

[70] Lim A, Gupta N, Lim A, Hong W, Walker K (2020). Description of the effect of patient flow, junior doctor supervision and pandemic preparation on the ability of emergency physicians to provide direct patient care. Aust Health Rev..

[71] Matsuo T, Taki F, Kobayashi D, Jinta T, Suzuki C, Ayabe A (2021). Health care worker burnout after the first wave of the coronavirus disease 2019 (COVID-19) pandemic in Japan. J Occup Health.

[72] Mosteiro-Díaz MP, Baldonedo-Mosteiro M, Borges E, Baptista P, Queirós C, Sánchez-Zaballos M (2020). Presenteeism in nurses: comparative study of Spanish, Portuguese and Brazilian nurses. Int Nurs Rev..

[73] Noorashid N, Chin WL. Coping with covid-19: the resilience and transformation of community-based tourism in Brunei Darussalam. Sustainability (Switzerland). 2021;13(15).

[74] Novak H, Tadić I, Falamić S, Ortner HM (2021). Pharmacists’ role, work practices, and safety measures against COVID-19: a comparative study. J Am Pharm Assoc..

[75] Palmer LA, Rousculp MD, Johnston SS, Mahadevia PJ, Nichol KL (2010). Effect of influenza-like illness and other wintertime respiratory illnesses on worker productivity: the child and household influenza-illness and employee function (CHIEF) study. Vaccine..

[76] Richmond BK, Dean LS, Farrell TM (2020). The impact of the COVID-19 pandemic on surgical practice in the southeastern United States: results of a survey of the membership of the southeastern surgical congress. Am Surg..

[77] Schanzer DL, Zheng H, Gilmore J. Statistical estimates of absenteeism attributable to seasonal and pandemic influenza from the Canadian labour force survey. BMC Infect Dis. 2011;11. 10.1186/1471-2334-11-90 PMC3103439 21486453

[78] Slone H, Gutierrez A, Lutzky C, Zhu D, Hedriana H, Barrera JF, et al. Assessing the impact of COVID-19 on mental health providers in the southeastern United States. Psychiatry Res. 2021;302. 10.1016/j.psychres.2021.114055 PMC8362842 34144509

[79] Tilchin C, Dayton L, Latkin CA (2021). Socioeconomic factors associated with an intention to work while sick from COVID-19. J Occup Environ Med..

[80] Torá-Rocamora I, Delclos GL, Martínez JM, Jardí J, Alberti C, Manzanera R (2012). Occupational health impact of the 2009 H1N1 flu pandemic: surveillance of sickness absence. Occup Environ Med..

[81] Tsai Y, Zhou F, Kim IK (2014). The burden of influenza-like illness in the US workforce. Occup Med..

[82] Turnea ES, Neștian ȘA, Tiță SM, Vodă AI, Guță AL (2020). Dismissals and temporary leaves in Romanian companies in the context of low demand and cash flow problems during the COVID-19 economic lockdown. Sustainability (Switzerland)..

[83] Van Der Feltz-Cornelis CM, Varley D, Allgar VL, de Beurs E (2020). Workplace stress, Presenteeism, absenteeism, and resilience Amongst University staff and students in the COVID-19 lockdown. Front Psych..

[84] van der Merwe P, Saayman A, Jacobs C. Assessing the economic impact of COVID-19 on the private wildlife industry of South Africa. Glob Ecol Conserv. 2021;28. 10.1016/j.gecco.2021.e01633 PMC8440124 34541259

[85] Van Wormer JJ, King JP, Gajewski A, McLean HQ, Belongia EA (2017). Influenza and workplace productivity loss in working adults. J Occup Environ Med..

[86] Webster A, Khorana S, Pastore F (2021). The effects of COVID-19 on employment. Labour Markets and Gender Equality in Central America.

[87] Widodo AW, Xavier C, Wibisono MR, Murti NMDA, Putra TP, Gunawan FE, et al. The impact of job stress on employee productivity during Covid-19 pandemic at the aviation industry. IOP Conf Ser. 2021;794:012084.

[88] Yohannes K, Roche P, Spencer J, Hampson A (2003). Annual report of the National Influenza Surveillance Scheme, 2002. Commun Dis Intell..

[89] Zaffina S, Gilardi F, Rizzo C, Sannino S, Brugaletta R, Santoro A (2019). Seasonal influenza vaccination and absenteeism in health-care workers in two subsequent influenza seasons (2016/17 and 2017/18) in an Italian pediatric hospital. Expert Rev Vaccines..

[90] Bhuiyan TR, Er AC, N. M, Pereira JJ. (2021). The socioeconomic impact of climate-related hazards: flash flood impact assessment in Kuala Lumpur, Malaysia. Nat Hazards.

[91] Phua KH (2005). A model for the costing of infectious disease epidemics.

[92] Suh M, Kang DR, Lee DH, Choi YJ, Tchoe B, Nam CM (2013). Socioeconomic burden of influenza in the Republic of Korea, 2007-2010. PLoS One.

[93] Richards F, Kodjamanova P, Chen X, Li N, Atanasov P, Bennetts L (2022). Economic burden of COVID-19: a systematic review. ClinicoEcon Outcomes Res..

[94] Feehan J (2021). Is COVID-19 the worst pandemic?. Maturitas..

[95] Daniels S, Wei H, Han Y, Catt H, Denning DW, Hall I (2021). Risk factors associated with respiratory infectious disease-related presenteeism: a rapid review. BMC Public Health.

[96] Smith AP, Thomas M, Brockman P, Kent J, Nicholson KG (1993). Effect of influenza B virus infection on human performance. BMJ..

[97] Du W, Wang G (2020). Indoor air pollution was nonnegligible during COVID-19 lockdown. Aerosol Air Qual Res..

[98] Shreta R. The economic impact of COVID-192020 12. 2022. Available from: https://www.tropicalmedicine.ox.ac.uk/news/the-economic-impact-of-covid-19. Accessed 9 Jan 2023.

[99] ILO monitor (2020). Covid-19 and the world of work.

[100] Lee A, Cho J (2016). The impact of epidemics on labor market: identifying victims of the Middle East respiratory syndrome in the Korean labor market. Int J Equity Health.

[101] Pak A, Adegboye OA, Adekunle AI, Rahman KM, McBryde ES, Eisen DP (2020). Economic consequences of the COVID-19 outbreak: the need for epidemic preparedness. Front Public Health.

[102] Xiang S, Rasool S, Hang Y, Javid K, Javed T, Artene AE (2021). The effect of COVID-19 pandemic on service sector sustainability and growth. Front Psychol..

[103] Tangcharoensathien V (2021). Are overwhelmed health systems an inevitable consequence of covid-19? Experiences from China, Thailand, and New York state. BMJ..

[104] Begley S. Flu-conomics: The next pandemic could trigger global recession. Reuters. 2013. Available from: https://www.reuters.com/article/us-reutersmagazine-davos-flu-economy-idUSBRE90K0F820130121. Accessed 9 Jan 2023.

[105] Rassy D, Smith RD (2013). The economic impact of H1N1 on Mexico's tourist and pork sectors. Health Econ..

[106] Lee C, Ki M (2015). Strengthening epidemiologic investigation of infectious diseases in Korea: lessons from the Middle East respiratory syndrome outbreak. Epidemiol Health.

[107] Abbas J, Mubeen R, Iorember PT, Raza S, Mamirkulova G (2021). Exploring the impact of COVID-19 on tourism: transformational potential and implications for a sustainable recovery of the travel and leisure industry. Curr Res Behav Sci..

[108] Baldwin RE, Tomiura E (2020). Thinking ahead about the trade impact of COVID-19. Economics in the time of COVID-19.

[109] McKibbin WJ (2009). The swine flu outbreak and its global economic impact.

[110] Lee JW, McKibbin WJ, Knobler S, Mahmoud A, Lemon S, Mack A, Sivitz L, Oberholtzer K (2004). Estimating the global economic costs of SARS. Learning from SARS: preparing for the next disease outbreak — workshop summary.

[111] United Nations. COVID-19 to slash global economic output by $8.5 trillion over next two years New York, United States. United Nations. 2020. Available from: https://www.un.org/en/desa/covid-19-slash-global-economic-output-85-trillion-over-next-two-years. Accessed 9 Jan 2023.

[112] Fan VY, Jamison DT, Summers LH (2018). Pandemic risk: how large are the expected losses?. Bull World Health Organ..

[113] The World Bank. COVID-19 to plunge global economy into worst recession since world war II [press release, June 8 2020]. The World Bank. 2020.

[114] Inayat A, Shahbaz A (2020). Why may COVID-19 overwhelm low-income countries like Pakistan?. Disaster Med Public Health Prep..

[115] Hsiang S, Allen D, Annan-Phan S, Bell K, Bolliger I, Chong T (2020). The effect of large-scale anti-contagion policies on the COVID-19 pandemic. Nature..

[116] Bloom DE, Cadarette D, Sevilla JP (2018). Epidemics and economics Washington, United States of America.

[117] Josephson A, Kilic T, Michler JD (2021). Socioeconomic impacts of COVID-19 in low-income countries. Nat Hum Behav..

[118] Guest JL, Rio CD, Sanchez T (2020). The three steps needed to end the COVID-19 pandemic: bold public health leadership, rapid innovations, and courageous political will. JMIR Public Health Surveill..

[119] Elden NMK, Mandil AMA, Hegazy AA, Nagy N, Mabry RM, Khairy WA (2022). Health innovations in response to the COVID-19 pandemic: perspectives from the eastern Mediterranean region. J Public Health (Oxford)..

