# Supplementary material for: Socioeconomic impacts of airborne and droplet-borne infectious diseases on industries: a systematic review
Source: BMC Infect Dis. 2024 Jan 16;24:93. doi: 10.1186/s12879-024-08993-y (PMC10792877; doi:10.1186/s12879-024-08993-y)
Supplement: Supplementary file 1 — Additional file 1. [file 12879_2024_8993_MOESM1_ESM.docx]

| **Supplementary Document 1**  **Search strategy** | | |
| --- | --- | --- |
| **Database** | **Search Terms** | **Search Field Descriptor** |
| PubMed | (“socioeconomic impact” OR “safety and health impact” OR “understaffing” OR “reduced workforce” OR “service disruption” OR “financial loss” OR “increased expenses” OR “productivity” OR “absenteeism” OR “presenteeism” OR “mental disorder” OR “burnout”)  AND  (infection OR “infectious disease” OR “communicable disease” OR outbreak OR epidemic OR pandemic OR COVID-19 OR SARS OR MERS OR coronavirus OR influenza OR adenovirus OR enterovirus OR rotavirus OR measles OR mumps OR smallpox OR tuberculosis OR diphtheria OR anthrax OR legionellosis OR meningococcus OR pneumococcus OR Bordetella OR aspergillosis OR blastomycosis OR cryptococcosis)  AND  (industr* OR workplace OR organization OR organisation OR compan*) | Title and abstract |
| Scopus |  | Titles, abstract and keywords |
| Web of Science |  | Abstract |
| IDEAS/REPEC | ("socioeconomic impact" OR "social impact" OR "economic impact")  AND  (infection OR “infectious disease” OR “communicable disease” OR outbreak OR epidemic OR pandemic OR COVID-19 OR SARS OR MERS OR coronavirus OR influenza OR adenovirus OR enterovirus OR rotavirus OR measles OR mumps OR smallpox OR tuberculosis OR diphtheria OR anthrax OR legionellosis OR meningococcus OR pneumococcus OR Bordetella OR aspergillosis OR blastomycosis OR cryptococcosis)  AND  (industr* OR workplace OR organization OR organisation OR compan*) | All |
| OSHLINE (via OSH References) | impact  AND  (infection OR “infectious disease” OR “communicable disease” OR outbreak OR epidemic OR pandemic OR COVID-19 OR SARS OR MERS OR coronavirus OR influenza OR adenovirus OR enterovirus OR rotavirus OR measles OR mumps OR smallpox OR tuberculosis OR diphtheria OR anthrax OR legionellosis OR meningococcus OR pneumococcus OR Bordetella OR aspergillosis OR blastomycosis OR cryptococcosis)  AND  (industr* OR workplace OR organization OR organisation OR compan*) | Subject |
| HSELINE (via OSH References) |  |  |
| NIOSHTIC-2 (via OSH References) |  |  |
