# Supplementary material for: Socioeconomic impacts of airborne and droplet-borne infectious diseases on industries: a systematic review
Source: BMC Infect Dis. 2024 Jan 16;24:93. doi: 10.1186/s12879-024-08993-y (PMC10792877; doi:10.1186/s12879-024-08993-y)
Supplement: Supplementary file 2 — Additional file 2. [file 12879_2024_8993_MOESM2_ESM.docx]

**Supplementary Document 2**

**Appendix A. Cincinnati Children’s Hospital Medical Center (CCHMC) Table of Evidence Levels**

**Appendix B. Joanna Briggs Institute (JBI) tools**

JBI Critical Appraisal Checklist for
analytical cross sectional studies

Reviewer ______________________________________ Date_______________________________

Author_______________________________________ Year_________ Record Number_________

|  | Yes | No | Unclear | Not applicable |
| --- | --- | --- | --- | --- |
| 1. Were the criteria for inclusion in the sample clearly defined? | □ | □ | □ | □ |
| 1. Were the study subjects and the setting described in detail? | □ | □ | □ | □ |
| 1. Was the exposure measured in a valid and reliable way? | □ | □ | □ | □ |
| 1. Were objective, standard criteria used for measurement of the condition? | □ | □ | □ | □ |
| 1. Were confounding factors identified? | □ | □ | □ | □ |
| 1. Were strategies to deal with confounding factors stated? | □ | □ | □ | □ |
| 1. Were the outcomes measured in a valid and reliable way? | □ | □ | □ | □ |
| 1. Was appropriate statistical analysis used? | □ | □ | □ | □ |

Overall appraisal: Include □ Exclude □ Seek further info □

Comments (Including reason for exclusion)

________________________________________________________________________________________________________________________________________________________________________________________________________________________________________________________________________________________________

JBI Critical Appraisal Checklist for
case control studies

Reviewer ______________________________________ Date_______________________________

Author_______________________________________ Year_________ Record Number_________

|  | Yes | No | Unclear | Not applicable |
| --- | --- | --- | --- | --- |
| 1. Were the groups comparable other than the presence of disease in cases or the absence of disease in controls? | □ | □ | □ | □ |
| 1. Were cases and controls matched appropriately? | □ | □ | □ | □ |
| 1. Were the same criteria used for identification of cases and controls? | □ | □ | □ | □ |
| 1. Was exposure measured in a standard, valid and reliable way? | □ | □ | □ | □ |
| 1. Was exposure measured in the same way for cases and controls? | □ | □ | □ | □ |
| 1. Were confounding factors identified? | □ | □ | □ | □ |
| 1. Were strategies to deal with confounding factors stated? | □ | □ | □ | □ |
| 1. Were outcomes assessed in a standard, valid and reliable way for cases and controls? | □ | □ | □ | □ |
| 1. Was the exposure period of interest long enough to be meaningful? | □ | □ | □ | □ |
| 1. Was appropriate statistical analysis used? | □ | □ | □ | □ |

Overall appraisal: Include □ Exclude □ Seek further info □

Comments (Including reason for exclusion)

________________________________________________________________________________________________________________________________________________________________________________________________________________________________________________________________________________________________

JBI Critical Appraisal Checklist for cohort studies

Reviewer ______________________________________ Date_______________________________

Author_______________________________________ Year_________ Record Number_________

|  | Yes | No | Unclear | Not applicable |
| --- | --- | --- | --- | --- |
| 1. Were the two groups similar and recruited from the same population? | □ | □ | □ | □ |
| 1. Were the exposures measured similarly to assign people to both exposed and unexposed groups? | □ | □ | □ | □ |
| 1. Was the exposure measured in a valid and reliable way? | □ | □ | □ | □ |
| 1. Were confounding factors identified? | □ | □ | □ | □ |
| 1. Were strategies to deal with confounding factors stated? | □ | □ | □ | □ |
| 1. Were the groups/participants free of the outcome at the start of the study (or at the moment of exposure)? | □ | □ | □ | □ |
| 1. Were the outcomes measured in a valid and reliable way? | □ | □ | □ | □ |
| 1. Was the follow up time reported and sufficient to be long enough for outcomes to occur? | □ | □ | □ | □ |
| 1. Was follow up complete, and if not, were the reasons to loss to follow up described and explored? | □ | □ | □ | □ |
| 1. Were strategies to address incomplete follow up utilized? | □ | □ | □ | □ |
| 1. Was appropriate statistical analysis used? | □ | □ | □ | □ |

Overall appraisal: Include □ Exclude □ Seek further info □

Comments (Including reason for exclusion)

________________________________________________________________________________________________________________________________________________________________________________________________

JBI Critical Appraisal Checklist for
studies reporting prevalence data

Reviewer ______________________________________ Date_______________________________

Author_______________________________________ Year_________ Record Number_________

|  | Yes | No | Unclear | Not applicable |
| --- | --- | --- | --- | --- |
| 1. Was the sample frame appropriate to address the target population? | □ | □ | □ | □ |
| 1. Were study participants sampled in an appropriate way? | □ | □ | □ | □ |
| 1. Was the sample size adequate? | □ | □ | □ | □ |
| 1. Were the study subjects and the setting described in detail? | □ | □ | □ | □ |
| 1. Was the data analysis conducted with sufficient coverage of the identified sample? | □ | □ | □ | □ |
| 1. Were valid methods used for the identification of the condition? | □ | □ | □ | □ |
| 1. Was the condition measured in a standard, reliable way for all participants? | □ | □ | □ | □ |
| 1. Was there appropriate statistical analysis? | □ | □ | □ | □ |
| 1. Was the response rate adequate, and if not, was the low response rate managed appropriately? | □ | □ | □ | □ |

Overall appraisal: Include □ Exclude □ Seek further info □

Comments (Including reason for exclusion)

________________________________________________________________________________________________________________________________________________________________________________________________

JBI Critical Appraisal Checklist for
Qualitative Research

Reviewer ______________________________________ Date_______________________________

Author_______________________________________ Year_________ Record Number_________

|  | Yes | No | Unclear | Not applicable |
| --- | --- | --- | --- | --- |
| 1. Is there congruity between the stated philosophical perspective and the research methodology? | □ | □ | □ | □ |
| 1. Is there congruity between the research methodology and the research question or objectives? | □ | □ | □ | □ |
| 1. Is there congruity between the research methodology and the methods used to collect data? | □ | □ | □ | □ |
| 1. Is there congruity between the research methodology and the representation and analysis of data? | □ | □ | □ | □ |
| 1. Is there congruity between the research methodology and the interpretation of results? | □ | □ | □ | □ |
| 1. Is there a statement locating the researcher culturally or theoretically? | □ | □ | □ | □ |
| 1. Is the influence of the researcher on the research, and vice- versa, addressed? | □ | □ | □ | □ |
| 1. Are participants, and their voices, adequately represented? | □ | □ | □ | □ |
| 1. Is the research ethical according to current criteria or, for recent studies, and is there evidence of ethical approval by an appropriate body? | □ | □ | □ | □ |
| 1. Do the conclusions drawn in the research report flow from the analysis, or interpretation, of the data? | □ | □ | □ | □ |

Overall appraisal: Include □ Exclude □ Seek further info □

Comments (Including reason for exclusion)

________________________________________________________________________________________________________________________________________________________________________________________________

**Appendix C. Center of Evidence Based Management (CEBMa) case study critical appraisal checklist**
